# A new bioluminescent reporter system to study the biodistribution of systematically injected tumor-derived bioluminescent extracellular vesicles in mice

**DOI:** 10.18632/oncotarget.22493

**Published:** 2017-11-18

**Authors:** Prakash Gangadaran, Xiu Juan Li, Ho Won Lee, Ji Min Oh, Senthilkumar Kalimuthu, Ramya Lakshmi Rajendran, Seung Hyun Son, Se Hwan Baek, Thoudam Debraj Singh, Liya Zhu, Shin Young Jeong, Sang-Woo Lee, Jaetae Lee, Byeong-Cheol Ahn

**Affiliations:** ^1^ Department of Nuclear Medicine, Kyungpook National University School of Medicine and Hospital, Daegu 700-721, Republic of Korea; ^2^ Department of Medical Oncology, All India Institute of Medical Sciences (AIIMS), Ansari Nagar, New Delhi 110029, India

**Keywords:** imaging, extracellular vesicle, bioluminescence, in vivo biodistribution, thyroid cancer

## Abstract

*In vivo* biodistribution and fate of extracellular vesicles (EVs) are still largely unknown and require reliable *in vivo* tracking techniques. In this study, *in vivo* bioluminescence imaging (BLI) using Renilla luciferase (Rluc) was developed and applied to monitoring of EVs derived from thyroid cancer (CAL-62 cells) and breast cancer (MDA-MB-231) in nude mice after intravenous administration and was compared with a dye-based labeling method for EV derived from CAL-62 cells. The EVs were successfully labeled with Rluc and visualized by BLI in mice. *In vivo* distribution of the EVs, as measured by BLI, was consistent with the results of *ex vivo* organ analysis. EV-CAL-62/Rluc showed strong signals at lung followed by liver, spleen & kidney (*P* < 0.05). EV-MDA-MB-231/Rluc showed strong signals at liver followed by lung, spleen & kidney (*P* < 0.05). EV-CAL-62/Rluc and EV-MDA-MB-231/Rluc stayed in animal till day 9 and 3, respectively; showed a differential distribution. Spontaneous EV-CAL-62/Rluc shown distributed mostly to lung followed by liver, spleen & kidney. The new BLI system used to show spontaneous distribution of EV-CAL-62/Rluc in subcutaneous CAL-62/Rluc bearing mice. Dye (DiR)-labeled EV-CAL-62/Rluc showed a different distribution *in vivo* & *ex vivo* compared to EV-CAL-62/Rluc. Fluorescent signals were predominately detected in the liver (*P* < 0.05) and spleen (*P* < 0.05) regions. The bioluminescent EVs developed in this study may be used for monitoring of EVs *in vivo*. This novel reporter-imaging approach to visualization of EVs in real time is expected to pave the way for monitoring of EVs in EV-based treatments.

## INTRODUCTION

Extracellular vesicles (EVs) are nano-sized membrane-bound vesicles that are released from cells into the extracellular space. EVs include microvesicles (diameter 50–400 nm) that are produced by budding of the plasma membrane [1] and exosomes (40–100 nm), which are released into the extracellular milieu upon fusion of multi-vesicular bodies with the plasma membrane [2–4] and are capable of carrying proteins, lipids, mRNA, miRNA, and even extra-chromosomal DNA [5–10]. Aside from healthy/nonmalignant cells, tumor cells also release EVs into their microenvironment. These are known as tumor-derived EVs [11, 12]. The role of EVs as mediators of cell-to-cell communication is being extensively studied [13–15]. Recent studies have shown successful monitoring of EVs and their potential as *in vivo* delivery vehicles for therapeutic agents such as chemotherapeutics, nucleic acids, and suicide-inducing genes/proteins [16–18]. EVs reflect the function of their parent cells because the contents of the vesicles include miRNA, mRNA, proteins, and membranes derived from the parent cell [19]. Accordingly, tumor-derived EVs may function similarly to tumor cells. For example, exosomes released from breast cancer carcinomas stimulate cell movement, leading to metastasis [20, 21]. Recent studies showed that tumor-derived EVs promote endothelial cell migration during angiogenesis in the tumor microenvironment via ERK1/2 and JNK signaling pathways [12]. Additionally, microRNA miR-122 of tumor-derived EV's can reprogram systemic energy metabolism to facilitate disease progression [21]. EVs from bile duct carcinomas mediate interactions between the tumor and mesenchymal stem cells and modulate tumor cell proliferation [22]. Numerous studies have shown that tumor-derived EVs transfer oncogenic activity, thus promoting tumor progression [23, 24]. Hence, an increasing body of evidence suggests that EVs can promote tumor development via numerous mechanisms, and it is therefore necessary to elucidate the distribution and clearance of tumor-derived EV. There has been a recent increase in the number of studies on EVs, indicating that this field is expanding rapidly [25]. Despite intensive research in this area, only a few studies have analyzed EV biodistribution in *in vivo* animal models [26–32]. A number of studies have shown biodistribution of EVs in various cancers; however, to our knowledge, the distribution of EVs in thyroid cancer has not been studied yet. *In vivo* trafficking of EVs in animal models is not well understood. Bioluminescence imaging (BLI) is a powerful method for cell tracking in small animals (such as mice) over time, without requiring the subject to be euthanized [33, 34]. Research involving BLI for monitoring EV distribution without exogenous dyes is very limited [29, 31, 35, 36]. An exogenous dye produces a nonspecific signal due to the long half-life of the fluorescent dye and its resistance to degradation. One of the main considerations when a fluorescent dye is used to label the cell membrane is that the dye can be released into the tissue; this situation can lead to the production of non-membrane-associated signals [32, 37, 38] In addition, labeling of EVs with exogenous signaling agents can result in changes to the characteristics of EVs, due to the labeling procedures used. To date, there has been a steadily growing number of studies on dye-based labeling of EVs, but it is necessary to ensure that the results observed reflect reality. Therefore, the necessity of an alternative to dye-based methods is inevitable. The distribution pattern of EV using a dye-based labeling method because it may provide interesting comparative data. We selected *Renilla* luciferase (Rluc), which is a cofactorless, single-subunit, blue-light-emitting luciferase isolated from the marine anthozoan *Renilla reniformis* (RLUC, E.C. number 1.13.12.5, luciferin-2-monooxygenase, decarboxylating) [39]. Using molecular oxygen, Rluc catalyzes oxidative decarboxylation. Relaxation of the electronically excited coelenteramide reaction product is accompanied by emission of a photon of blue light (∼470 nm) [40–42]. In the present study, we designed a highly sensitive bioluminescent EV reporter system that enables noninvasive *in vivo* imaging of EVs. In order to compare the distribution of EVs systemically delivered to mice, a near-infrared (NIR) dye, DiR which was used in previous studies [27, 32, 43] was also used for the EV labeling. We also used a human breast cancer cell line (MDA-MB-231) to show new bioluminescent reporter is really working. We analyzed the visualization of EVs in a mouse model using the newly developed bioluminescent EVs and DiR-labeled EVs during noninvasive real-time molecular imaging and studied the biodistribution and fate of tumor-derived EVs in the mouse model after an intravenous injection, as a function of time.

## RESULTS

### Generation of stable reporter gene expression in cancer cell line

In order to study the distribution of EVs derived from cancer cells using the imaging reporter system, Rluc was stably transduced into a human anaplastic thyroid cancer cell line CAL-62 cells (CAL-62/Rluc) and a human breast cancer cell line MDA-MB-231 via lentiviral delivery of the Rluc gene (Supplementary Figure 1A). The Rluc protein in cancer cells reacts with coelenterazine, and this interaction results in emission of light of wavelength between 475 and 480 nm. Thus, we evaluated the functional activity of Rluc using an *in vivo* optical imaging system in live CAL-62/Rluc, MDA-MB-231/Rluc and their respective parental cells, after addition of coelenterazine. The BLI signal was stronger in CAL-62/Rluc or MDA-MB-231/Rluc cells than in their parental cells and increased in a cell number-dependent manner (CAL-62/Rluc: Supplementary Figure 1B; *R*^2^ = 0.98; MDA-MB-231/Rluc: Supplementary Figure 1D; *R*^2^ = 0.97). Western blot analyses of lysates of CAL-62/Rluc or MDA-MB-231/Rluc Cells revealed a band of the expected size for Rluc at 37 kDa, but no band was observed for their parental cells. Furthermore, to analyze the gene expression of Rluc genes in CAL-62/Rluc or MDA-MB-231/Rluc cells, RT-PCR analysis was performed. Rluc gene expression was successfully detected in CAL-62/Rluc or MDA-MB-231/Rluc cells; however, this was not the case for parental CAL-62 or MDA-MB-231 cells (Supplementary Figure 1). Taken together, these results indicate that Rluc was stably expressed in the CAL-62/Rluc and MDA-MB-231/Rluc cells. These cells were used for subsequent isolation of EVs and other experiments.

### Isolation and characterization of EVs

**Figure 1:**
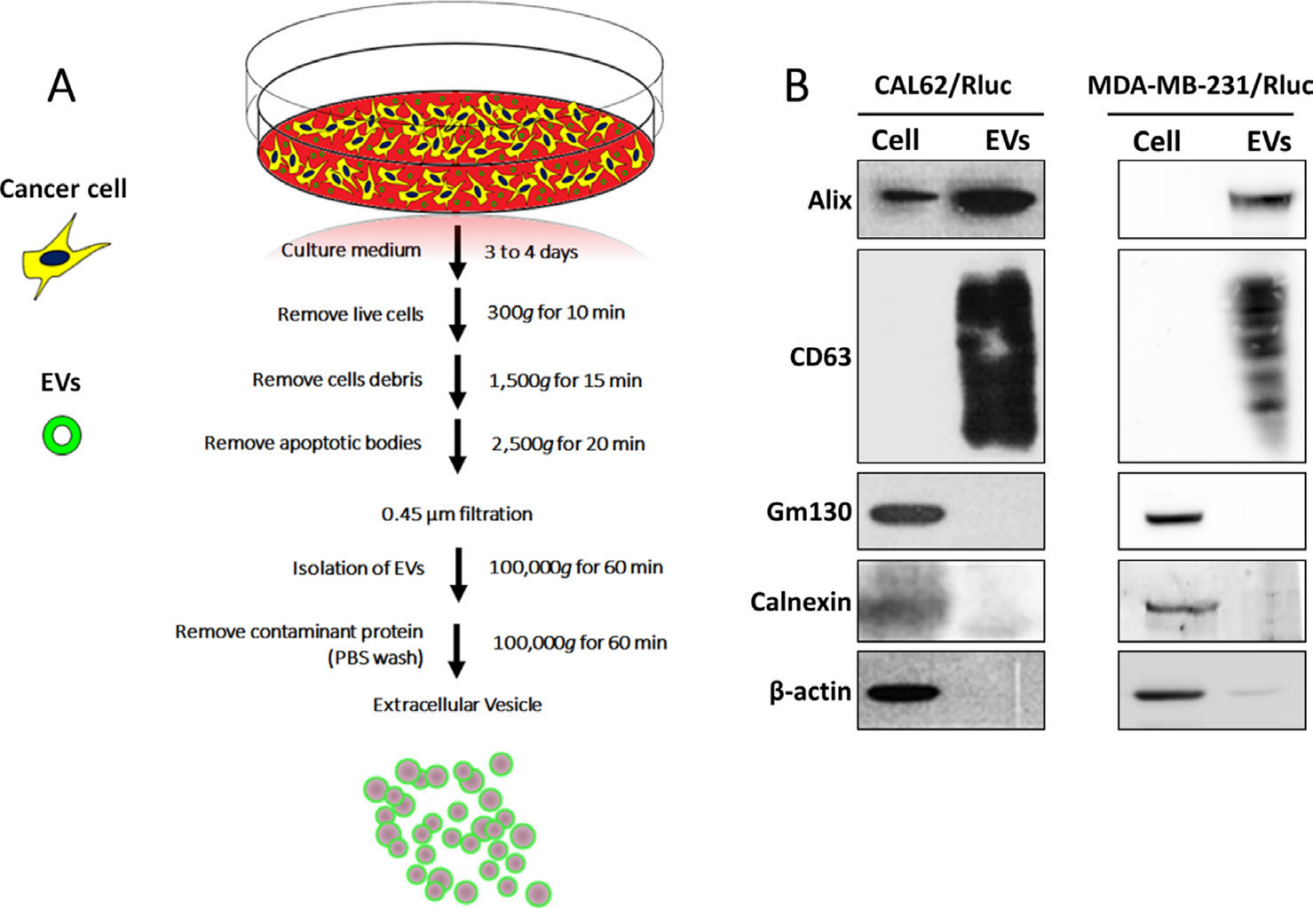
Isolation and characterization of EVs (**A**) A flow chart for the EV purification procedure based on ultracentrifugation. (**B**) Western blotting analysis of EVs. ALIX and CD63, EV marker proteins were detected by using anti-ALIX (97 kDa) and anti-CD63 (53 kDa) specific antibodies, respectively; GM-130 and Calnexin, EV negative marker proteins were detected by using anti-GM-130 (112 kDa) and anti-calnexin (90 kDa) specific antibodies, respectively; β-actin was used as a loading control.

**Figure 2:**
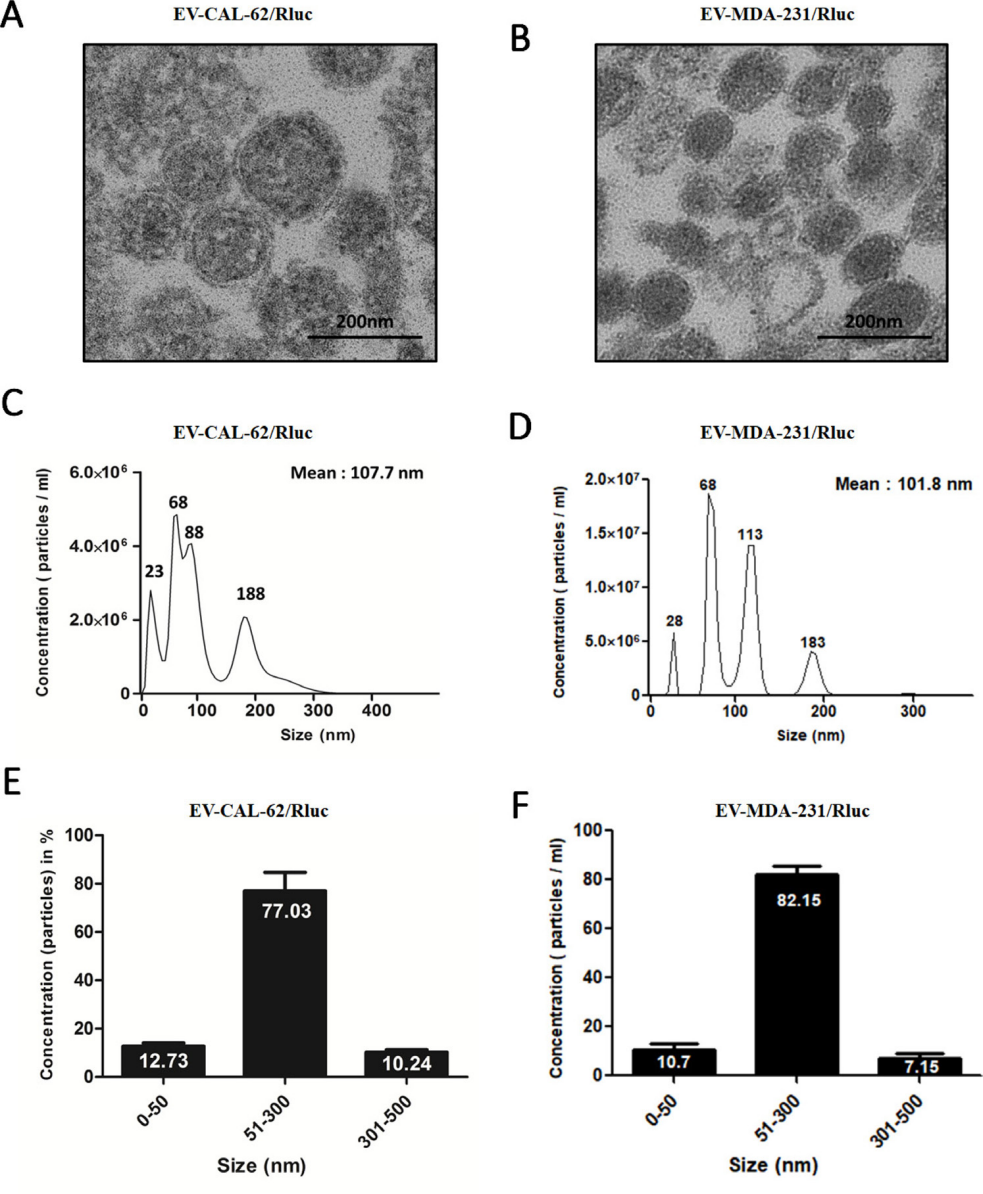
Typical characteristics of EVs and size distribution by NanoSight measurements (**A**, **B**) Electron-microscopic observation of EVs. EVs are diverse in their density and size, with a size range of 50 to 450 nm in diameter (scale bars, 200 nm). (**C**, **D**) Size distribution of EVs measured NanoSight. (**E**, **F**) Percentage distribution of EVs, shown as a bar diagram, as analyzed by NanoSight; Results shown in E, F represent mean ± SD from three independent experiments.

We isolated EVs from the conditioned medium obtained from CAL-62, CAL-62/Rluc, MDA-MB-231 and MDA-MB-231/Rluc cells by ultracentrifugation as described in Current Protocols in Cell Biology [44] with modifications (Figure 1A). The proteins from the isolated EV-CAL-62/Rluc and MDA-MB-231/Rluc (further called as MDA-231/Rluc) were initially analyzed for common EV markers by western blot after ultracentrifugation [45, 46]. Western blotting analysis showed that vesicle trafficking-related protein ALIX and tetraspanin protein CD63, which are frequently identified vesicular proteins, were present (Figure 1B). In contrast, Golgi apparatus (GM130) and endoplasmic reticulum (calnexin) markers were detected in cell lysates but not in EVs, indicating efficient enrichment of EVs from the culture media (Figure 1B). Moreover, we performed further analysis to confirm the shape and size of purified EVs, using transmission electron microscopy (TEM) and NanoSight analysis. The TEM analysis revealed a heterogeneous mixture of vesicles with a diameter ranging from 50 to 450 nm, approximately. The TEM showed predominantly intact vesicles with classical EV morphology. EV preparations showed spherical-shape distributions. (Figure 2A, 2B). NTA yielded a similar vesicle size distribution: from 23 to 500 nm with a mean size of 107.7 nm, from 28 to 450 nm with a mean size of 101.8 nm in EV-CAL-62/Rluc and MDA-231/Rluc respectively (Figure 2C, 2D). The EV size ranging from 51 to 300 nm represented 77% and 82 % of EV-CAL-62/Rluc and MDA-231/Rluc respectively (Figure 2E, 2F). As noted above, both the EVs were successfully isolated and characterized.

### EV-CAL-62/Rluc and EV-MDA-231/Rluc showed EV-specific Rluc activity *in vitro*

**Figure 3:**
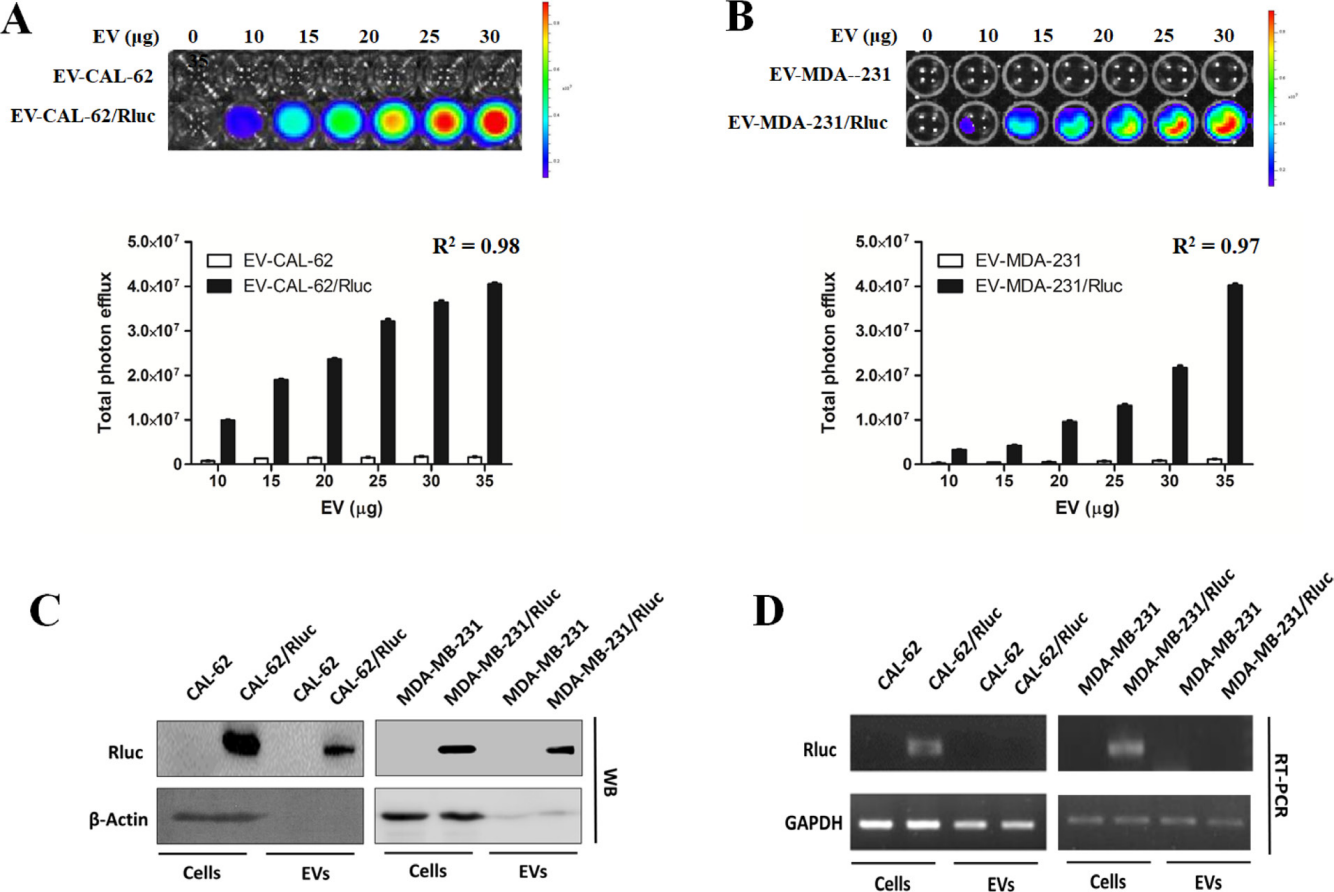
EV-CAL-62/Rluc showed EV-specific Rluc activity *in vitro* (**A**, **B**) Representative bioluminescent imaging of an *in vitro* luciferase assay in EVs from CAL-62, CAL-62/Rluc cells, MDA-MB-231 and MDA-MB-231/Rluc. Quantitative *in vitro* luciferase assay in EV's data are expressed as mean ± SD. (**C**) Western blot analysis of the Rluc protein in cells and EVs from CAL-62, CAL-62/Rluc & MDA-MB-231 and MDA-MB-231/Rluc cells, detected by means of Rluc-specific antibodies. β-Actin served as loading control. (**D**) RT-PCR analysis of Rluc mRNA in cells and EVs from CAL-62, CAL-62/Rluc & MDA-MB-231 and MDA-MB-231/Rluc cells. GAPDH was used as a loading control.

To confirm the presence of the Rluc reporter protein and to evaluate its luciferase activity in the EVs from CAL-62/Rluc and MDA-231/Rluc cells, EVs (EV-CAL-62/Rluc EV-CAL-62, EV-MDA-231/Rluc and EV-MDA-231) were prepared from the conditioned medium of Rluc expressing cells and parental cells by ultracentrifugation, using the procedure described in Figure 1A. We evaluated the luciferase activity of Rluc using IVIS Lumina II in EVs (EV-CAL-62/Rluc EV-CAL-62, EV-MDA-231/Rluc and EV-MDA-231), by addition of coelenterazine to the EVs. A very strong Rluc activity was detected in EV-CAL-62/Rluc and EV-MDA-231/Rluc, in contrast to the absence of bioluminescence in EV-CAL-62 and EV-MDA-231 (Figure 3A: *R*^2^ = 0.98; Figure 3B: *R*^2^ = 0.97 respectively). In addition, proteins collected from Rluc expressing cells and their respective EVs were further analyzed by western blotting and RT-PCR, which confirmed the presence of Rluc in EV-CAL-62/Rluc and EV-MDA-231/Rluc (Figure 3C). These results suggested that the Rluc reporter was present in the vesicle compartments of EV-CAL-62/Rluc and EV-MDA-231/Rluc. RT-PCR results revealed that mRNA of Rluc was not detectable in EV-CAL-62/Rluc and EV-MDA-231/Rluc (Figure 3D).

**Figure 4:**
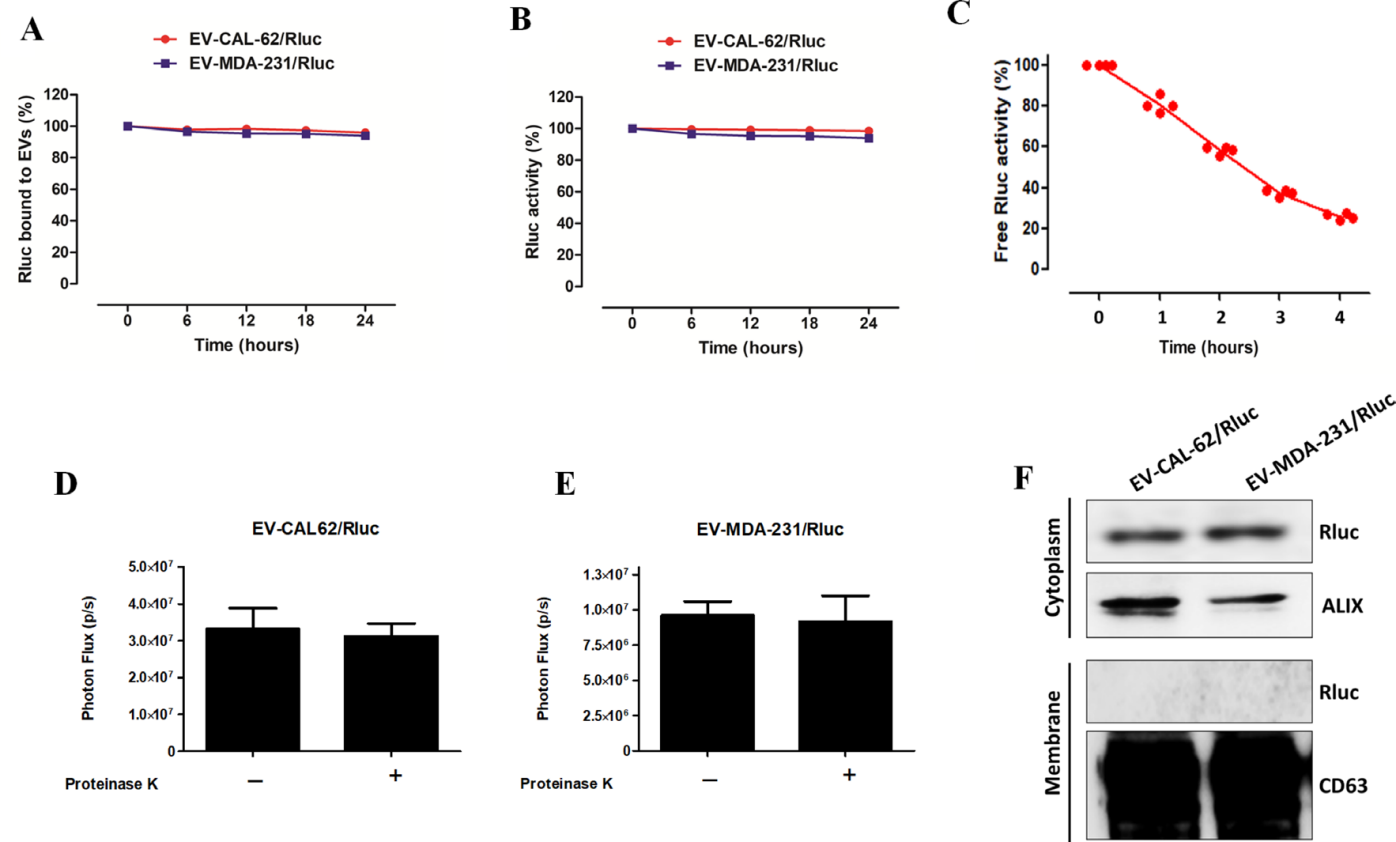
EV-CAL-62/Rluc and EV-MDA-231/Rluc showed high serum stability and Rluc protein incorporated inside the EVs (**A**) Rluc-binding capacity of EVs in serum. Time course of binding of Rluc in EV-CAL-62/Rluc and EV-MDA-231/Rluc at 37°C in 20% FBS/PBS buffer. Rluc activity was measured in serum. (**B**) Stability of Rluc activity of EV-CAL-62/Rluc and EV-MDA-231/Rluc in serum. Time course of stability of Rluc in EV at 37°C in 20% FBS/PBS buffer. (**C**) Stability of Rluc activity of Free Rluc protein in serum. Time course of stability of Rluc at 37°C in 20% FBS/PBS buffer. (**D**, **E**) EV-CAL-62/Rluc and EV-MDA-231/Rluc treated with proteinase K and without proteinase K and activity of Rluc was measured by IVIS. Data are expressed as mean ± SD. (**F**) Western blot analysis of the Rluc protein in cytosolic and membrane fraction of EV-CAL-62/Rluc & EV-MDA-231/Rluc, detected by means of Rluc-specific antibodies. Alix and CD63 served as loading control for cytosolic and membrane fraction respectively.

To evaluate the discharge of Rluc from EVs, EV-CAL-62/Rluc and EV-MDA-231/Rluc were incubated with 20% FBS in PBS and ultracentrifuged (after 6, 12, 18, and 24 hours) to remove the EVs. The Rluc activity was measured in the supernatant, and it showed only a very low level: less than 1.5% of Rluc activity from the initial level even after 24 hours (Figure 4A). This result indicated that little Rluc protein was discharged from the labeled EVs into the serum. EV-CAL-62/Rluc, EV-MDA-231/Rluc were incubated with 20% FBS in PBS (after 6, 12, 18, and 24 hours) to test the Rluc stability. Rluc activity marginally decreased to approximately 95% of the initial value after 24 hours of incubation (Figure 4B). Furthermore, we tested the recombinant Rluc (Free Rluc) protein stability in serum; our results suggest that Free Rluc protein BLI signals reduced 50% within 3 hours (Figure 4C). In addition we tested the presence of Rluc in the EVs membrane or inside the EV compartment, the proteinase K treatment to EVs showed no significant changes in the Rluc activity compare to no treatment EVs (Figure 4D, 4E). Furthermore, our western blot results clearly confirmed that Rluc present inside the EV compartment, as membrane fraction from EVs showed absence of Rluc protein and cytosolic fraction expressed an Rluc protein (Figure 4F). These results indicated that the novel bioluminescent reporter protein (Rluc) could be used to label EVs and was successfully developed.

### Rluc modulation within the cell does not alter the EV release, concentration, or morphology

We tested whether manipulation of Rluc expression in the cells may change the EV release, concentration, content, or morphology. EVs derived from the same number of CAL-62/Rluc, CAL-62, MDA-231/Rluc and MDA-231 cells were analyzed by NanoSight and TEM. Rluc overexpression in CAL-62 or MDA-231/Rluc cells had no effect on EV size, and average sizes of EV-CAL-62/Rluc and EV-CAL-62 were 107.7 and 108.6 nm, respectively and EV- MDA-231/Rluc and EV-MDA-231 were 101.8 and 99.6 nm, respectively (Supplementary Figure 2A–2D). Furthermore, the morphology of EVs derived from CAL-62 AND MDA-231/Rluc cells with modulated Rluc expression was identical to that of EVs produced by their respective parental cells (Supplementary Figure 2E–2H). Comparative analysis did not reveal any major alterations in EV release and morphology; therefore, the Rluc transduction into the CAL-62 and MDA-231/Rluc cell does not alter the characteristics of EVs. EV-CAL-62/Rluc and EV-CAL-62 concentrations were 7.77 × 10^7^ ± 6.11 × 10^6^ and 8.07 × 10^7^ ± 1.55 × 10^6^, respectively. EV-MDA-231/Rluc and EV-MDA-231 concentrations were 1.92 × 10^8^ ± 9.06 × 10^6^ and 1.82 × 10^8^ ± 9.08 × 10^6^, respectively (Supplementary Figure 2I–2J). This evidence strongly suggests that both populations indeed release the same concentrations of EVs. The Rluc expression (Photon Flux) in a single EVs of CAL-62/Rluc and MDA-MB-231/Rluc showed 0.035 ± 0.003 and 0.034 ± .009, respectively (Supplementary Figure 2K). Equivalent amounts of proteins extracted from CAL-62/Rluc or MDA-231/Rluc and CAL-62 or MDA-231 cells and their EVs were separated by SDS-PAGE and stained with Imperial^™^ protein stain (Supplementary Figure 2L). The protein samples isolated from the cells had a different profile as compared to EVs, in line with other reports [45, 46]. CAL-62/Rluc or MDA-231/Rluc and CAL-62 or MDA-231, respectively showed the same profile of proteins, and their respective EVs also had the same profile (Supplementary Figure 2L).

### *In vivo* noninvasive bioluminescent visualization of EV-CAL-62/Rluc and EV-MDA-231/Rluc biodistribution in nude mice (*In vivo* and *ex vivo*)

**Figure 5:**
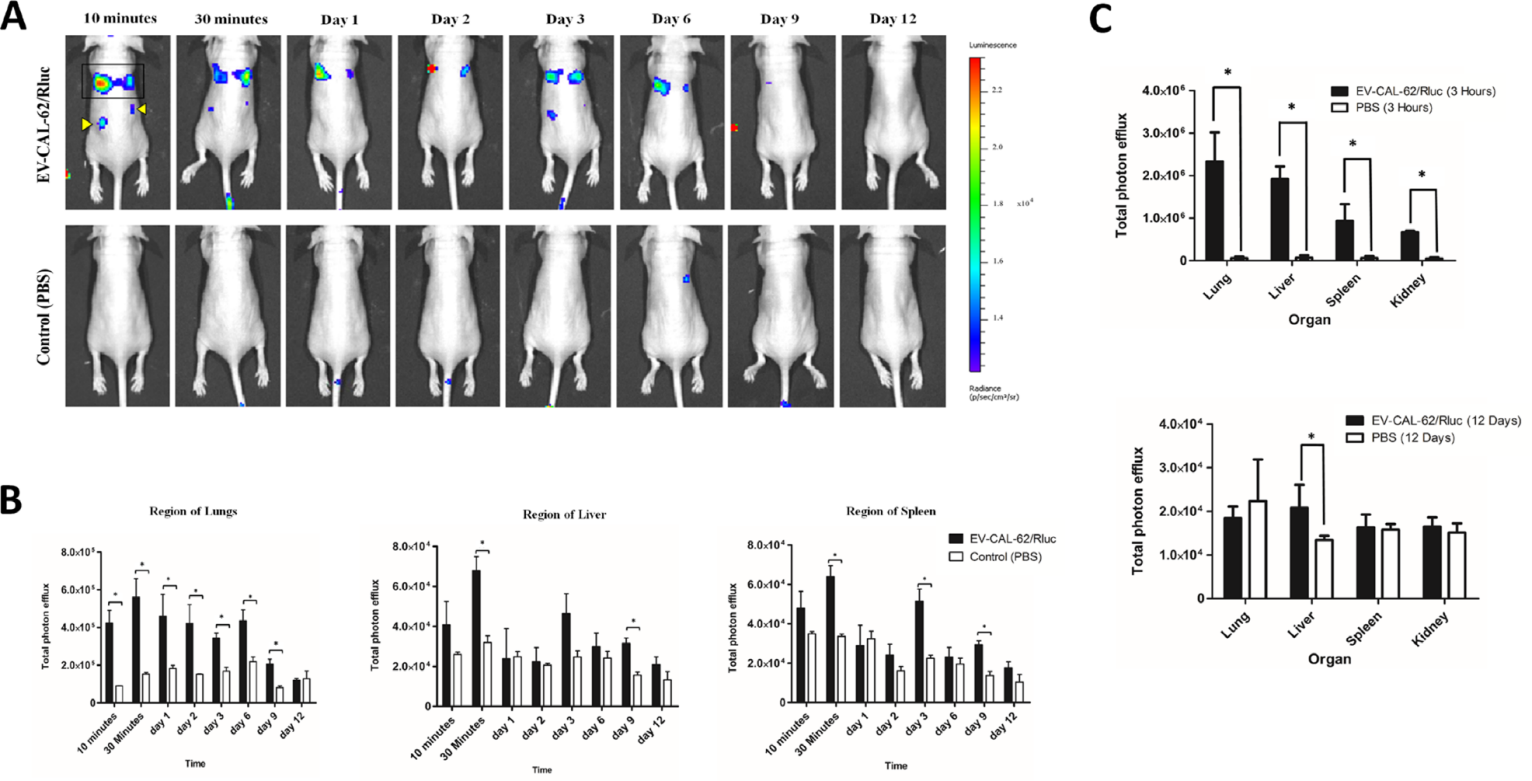
*In vivo* noninvasive bioluminescent visualization of EV-CAL-62/Rluc biodistribution in nude mice and organ distribution (**A**) Representative *in vivo* bioluminescent imaging (BLI) of EV-CAL-62/Rluc in nude mice. EV-CAL-62/Rluc or PBS (control) was administered via the tail vein. Coelenterazine was injected via the same route at 10 and 30 min and 1, 2, 3, 6, 9, and 12 days after initial administration to visualize EV-CAL-62/Rluc. Regions of the lung (four-sided box), liver (right arrowhead), and spleen (left arrowhead) are indicated in the animal (at 10 minutes). (**B**) Quantitation of EV-CAL-62/Rluc signal from regions corresponding to the lung, liver, and spleen after EV administration; the values are expressed as mean ± SD. ^*^P < 0.05 (Student's *t*-test). (**C**) Quantification of dissected organs of mice injected with EV-CAL-62/Rluc (*n* = 3) or PBS (*n* = 3). The mice were euthanized at 3 hours and 12 days after injection. Organs were harvested and lysed, and Rluc activity was measured. Bioluminescence quantification of lungs, liver, spleen, and kidneys at 3 hours and 12 days (EV-CAL-62/Rluc or PBS); the values are expressed as mean ± SD, ^*^P < 0.05, (Student's *t*-test).

In order to visualize and track the distribution of EVs after intravenous (i.v.) administration to nude mice, EV-CAL-62/Rluc or EV-MDA-231/Rluc was used. Naive mice were i.v. injected with 25 μg (protein) of EV-CAL-62/Rluc or EV-MDA-231/Rluc and control mice were injected with PBS. To assess the biodistribution of the EVs in mice by BLI, at 10 and 30 minutes and on Day 1, 2, 3, 6, 9, and 12 after EV-CAL-62/Rluc or EV-MDA-231/Rluc and PBS administration, coelenterazine was injected i.v. at the above-mentioned time points, and analyzed after injection, using an IVIS Lumina II. Systemically injected EV-CAL-62/Rluc were visualized in the mice within 10 minutes after the injection. BLI signals were detected primarily in the regions of lungs, liver, and spleen, and little or no signal was detected in control mice (Figure 5A). The signal intensity in the region of the liver, lungs, and spleen was quantified. At 10 and 30 minutes, a statistically significantly stronger BLI signal was detected in the region of lungs (*P* < 0.05; Figure 5B). The BLI signal revealed that the EVs initially undergo a rapid distribution phase. At 30 minutes, a statistically significant BLI signal was detected in the region of the liver (*P* < 0.05) and spleen (*P* < 0.05; Figure 5B). The BLI signals of EV-CAL-62/Rluc gradually decreased from 30 minutes (5.61 × 10^5^ ± 9.68 × 10^4^) to day 9, and no significant changes were seen on day 12 (1.20 × 10^5^ ± 1.03 × 10^4^). Furthermore, on Days 3 and 9, the BLI signal was suddenly found to decline in the region of the lungs (Figure 5B), but at the same time, the signal significantly increased in the liver region (*P* < 0.05) on Day 9, with a substantial increase observed on Day 3 (*P* = 0.064), and a significant increase in the spleen (*P* < 0.05) on Days 3 and 9. Nevertheless, most EV-CAL-62/Rluc were cleared from the animals by Day 12 post-injection, no signal was detected on Day 12 (Figure 5B). These EVs were probably cleared by both the liver and kidneys [47]. Our results clearly demonstrated that EV distribution can be visualized *in vivo* using the newly developed bioluminescent EVs with the reporter gene system; it is possible to analyze the biodistribution of EVs in real time. These experiments were designed to confirm the findings from *in vivo* EV-CAL-62/Rluc imaging and further analyze the organs from which the BLI imaging signal originally originated. To accurately identify the organ from which the BLI signal originated, organs were collected at two time points (3 hours and 12 days) after EV administration, to assess Rluc signals (Figure 5C and Supplementary Figure 3A). Significant BLI signals were detected only after 3 hours in mice injected with EV-CAL-62/Rluc, in lungs (*P* < 0.05), followed by liver (*P* < 0.05), spleen (*P* < 0.05), and kidneys (*P* < 0.05). The BLI signals of EVs were almost absent at 12 days after EV-CAL-62/Rluc injection, and at 3 hours or 12 days in PBS-injected mice (Figure 5C and Supplementary Figure 3A); however, the signal was detected at 12 days in the liver of EV-CAL-62/Rluc-injected mice (*P* < 0.05; Figure 5C and Supplementary Figure 3A).

**Figure 6:**
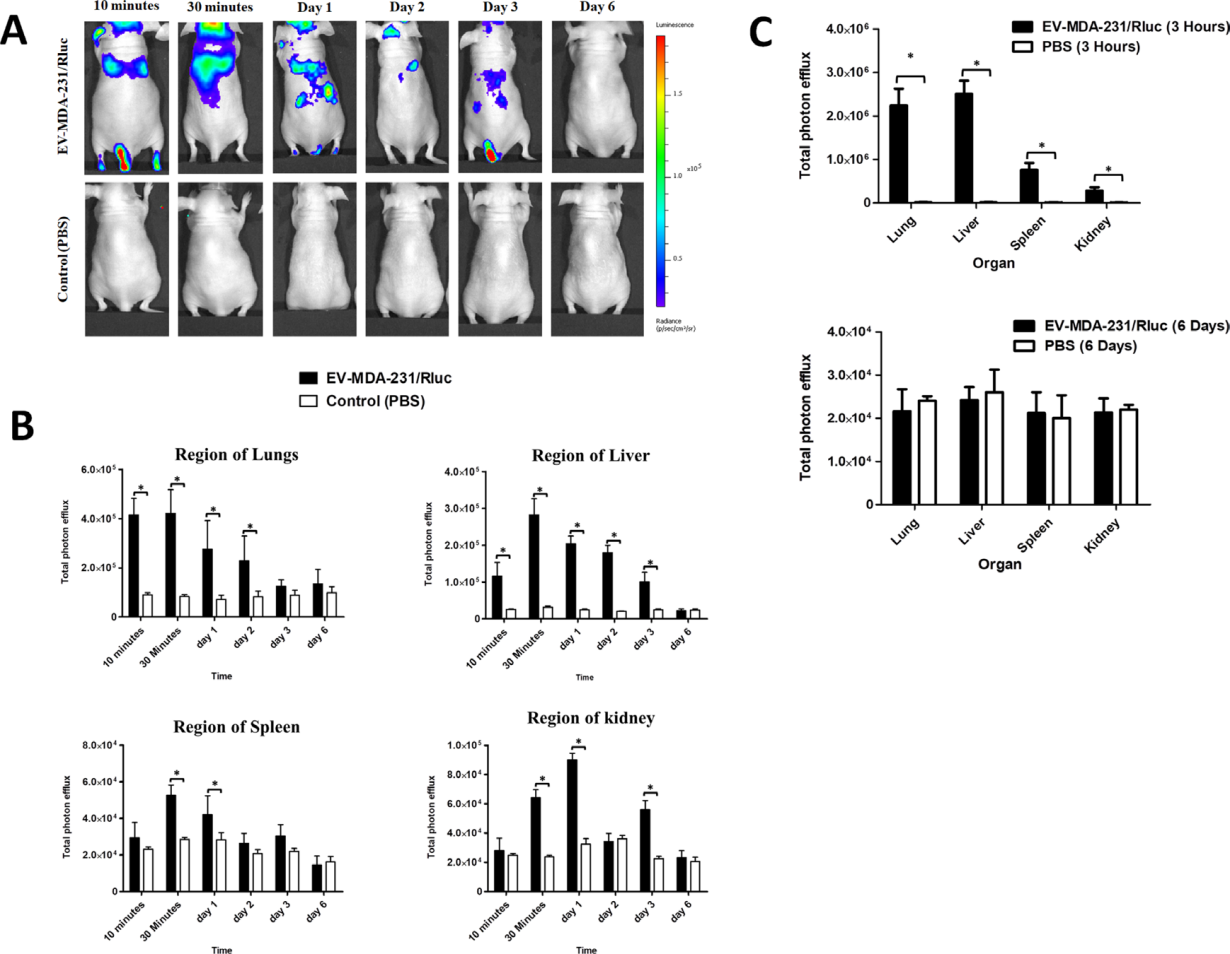
*In vivo* noninvasive bioluminescent visualization of EV-MDA-231/Rluc biodistribution in nude mice and organ distribution (**A**) Representative *in vivo* bioluminescent imaging (BLI) of EV-CAL-62/Rluc in nude mice. EV-MDA-231/Rluc or PBS (control) was administered via the tail vein. Coelenterazine was injected via the same route at 10 and 30 min and 1, 2, 3, and 6 days after initial administration to visualize EV-CAL-62/Rluc. (**B**) Quantitation of EV-MDA-231/Rluc signal from regions corresponding to the lung, liver, spleen and kidney after EV administration; the values are expressed as mean ± SD. ^*^P < 0.05 (Student's *t*-test). (**C**) Quantification of dissected organs of mice injected with EV-CAL-62/Rluc (*n* = 3) or PBS (*n* = 3). The mice were euthanized at 3 hours and 6 days after injection. Organs were harvested and lysed, and Rluc activity was measured. Bioluminescence quantification of lungs, liver, spleen, and kidneys at 3 hours and 6 days (EV-MDA-231/Rluc or PBS); the values are expressed as mean ± SD, ^*^P < 0.05, (Student's *t*-test).

Systemically injected EV-MDA-231/Rluc were visualized in the mice within 10 minutes after the injection. BLI signals were detected primarily in the regions of lungs, liver, spleen and kidney, and little or no signal was detected in control mice (Figure 6A). The signal intensity in the region of the liver, lungs, spleen and kidney was quantified. At 10, 30 minutes, day 1 and day 2, a significantly stronger BLI signal was detected in the region of lungs and liver (*P* < 0.05; Figure 6B). At 30 minutes and day 1, a significantly stronger BLI signal was detected in the region of the spleen (*P* < 0.05) and Kidney (*P* < 0.05; Figure 6B). The BLI signals of EV-MDA-231/Rluc gradually decreased from 30 minutes to day 3, and no significant changes were seen on day 6. Furthermore, on Days 3 the BLI signal was suddenly found to decline in the region of the lungs and liver (Figure 6B), but at the same time, the signal significantly increased in the kidney region (*P* < 0.05) on Day 3, shows that the EVs were cleared through kidney route. No significant signals were observed at day 6 in all the region of the organs. The most EV-MDA-231/Rluc were cleared from the animals by Day 6 post-injection (Figure 6B). These EVs were probably cleared by both the liver and kidneys [47]. Our results clearly demonstrated that the newly developed bioluminescent EVs with the reporter gene system can be used in various cells. To confirm the *in vivo* findings from imaging, further analyzed the exercised organs as mentioned above. The BLI signals detected at 3 hours, showed significantly stronger signals compare to control (*P* < 0.05; Figure 6C and Supplementary Figure 3B). The higher signals were detected in liver followed by lungs, spleen and kidney. At 6 hours, no statistically significant signals were detected in lungs, liver spleen and kidney (Figure 6C and Supplementary Figure 3B).

### Biodistribution of i.v. administered free Rluc protein in nude mice (*In vivo* and *ex vivo*)

**Figure 7:**
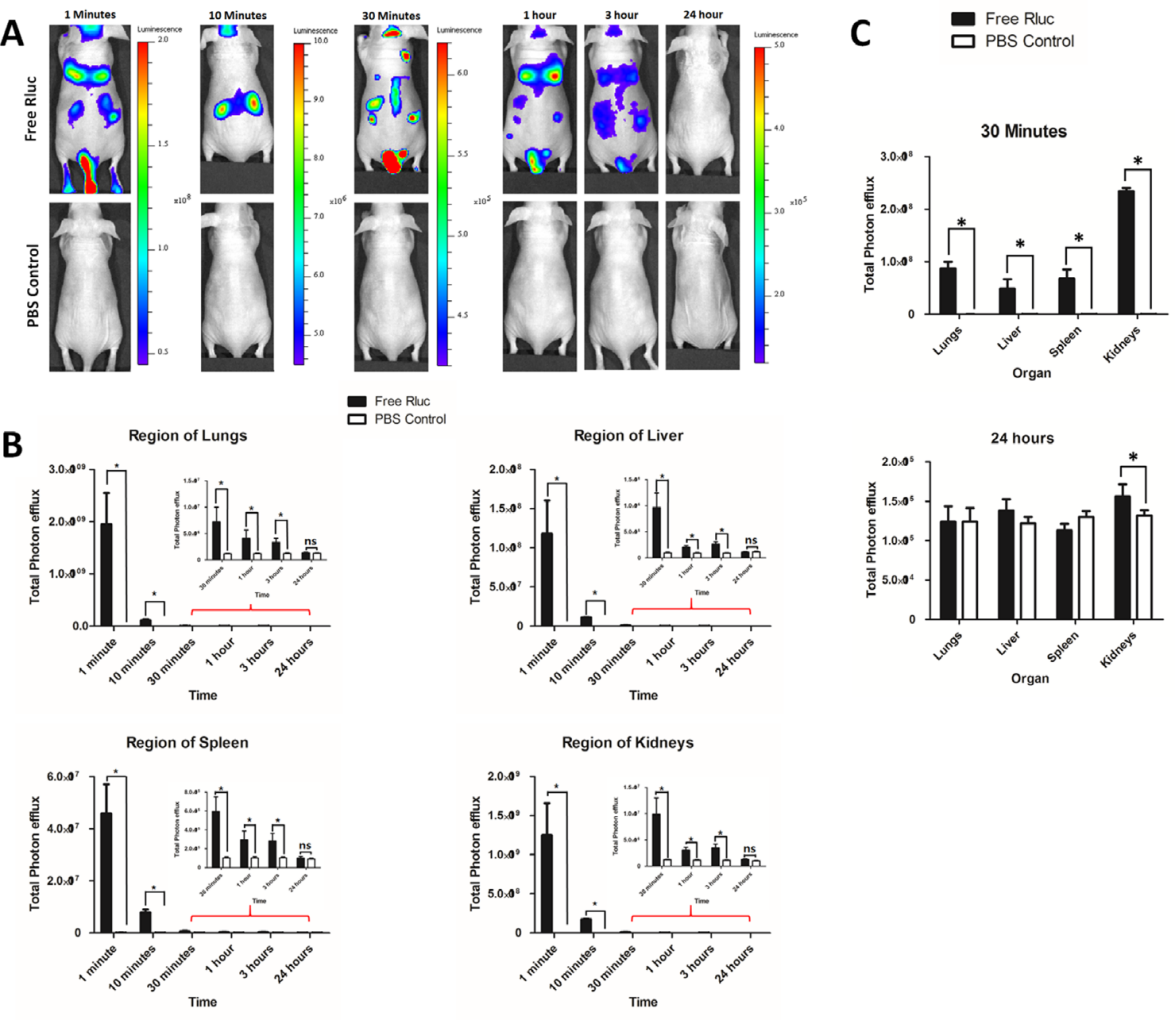
Biodistribution of i.v. administered free Rluc protein *in vivo* and organ distribution (**A**) Representative *in vivo* bioluminescent imaging (BLI) of mice injected with Free Rluc (*n* = 3) or PBS (*n* = 3). Coelenterazine was injected via the same route at 1, 10 and 30 min and 1, 3 and 24 hours after initial administration to visualize free Rluc protein. (**B**) Quantitation of Free Rluc signal from regions corresponding to the lung, liver, spleen and kidney after EV administration; the values are expressed as mean ± SD. ^*^P < 0.05 (Student's *t*-test). (**C**) Quantification of bioluminescent signals in lungs, liver, spleen, and kidneys at 3 minutes and 24 hours (Free Rluc or PBS). The values are expressed as mean ± SD, ^*^P < 0.05, (Student's *t*-test).

The biodistribution of free Rluc protein are presented in Figure 7A. Free Rluc protein signals were detected from 1 minute post injection. The BLI signals of Free Rluc rapidly decreased from 1 minute to 10 minutes about 43 folds (Region of all organs). BLI signals gradually decreased from 30 minutes to 3 hours. Twenty-four hours imaging showed no significant signal in mice compare to control (*P* > 0.05; Figure 7B). All time point no signals was detected in PBS group. A statistically significantly stronger BLI signal was detected in the region of lungs, liver, spleen and kidney compare to control (*P* < 0.05; Figure 7B). At 1 minute, stronger signal was detected in lungs and kidney; at 10 and 30 minutes, a stronger BLI signal was detected in the region of kidneys compare to other organs (Figure 7B). The distributions of the free Rluc protein and that of the labeled EVs (EV-CAL-62/Rluc or EV-MDA-231/Rluc) differ drastically. To confirm the *in vivo* findings from imaging, further analyzed the exercised organs as mentioned above. The BLI signals detected at 30 minutes, showed significantly stronger signals compare to control (*P* < 0.05; Figure 7C and Supplementary Figure 3C). The higher signals were detected in kidney followed by lungs, spleen and liver. At 24 hours, no statistically significant signals were detected in Lungs, Liver and Spleen. Whereas kidneys showed statically significant signals compare to control *(P* < 0.05; Figure 7C and Supplementary Figure 3C).

### *In vivo* noninvasive fluorescent visualization of EV-CAL-62/Rluc/DiR biodistribution in nude mice (*In vivo* and *ex vivo*)

**Figure 8:**
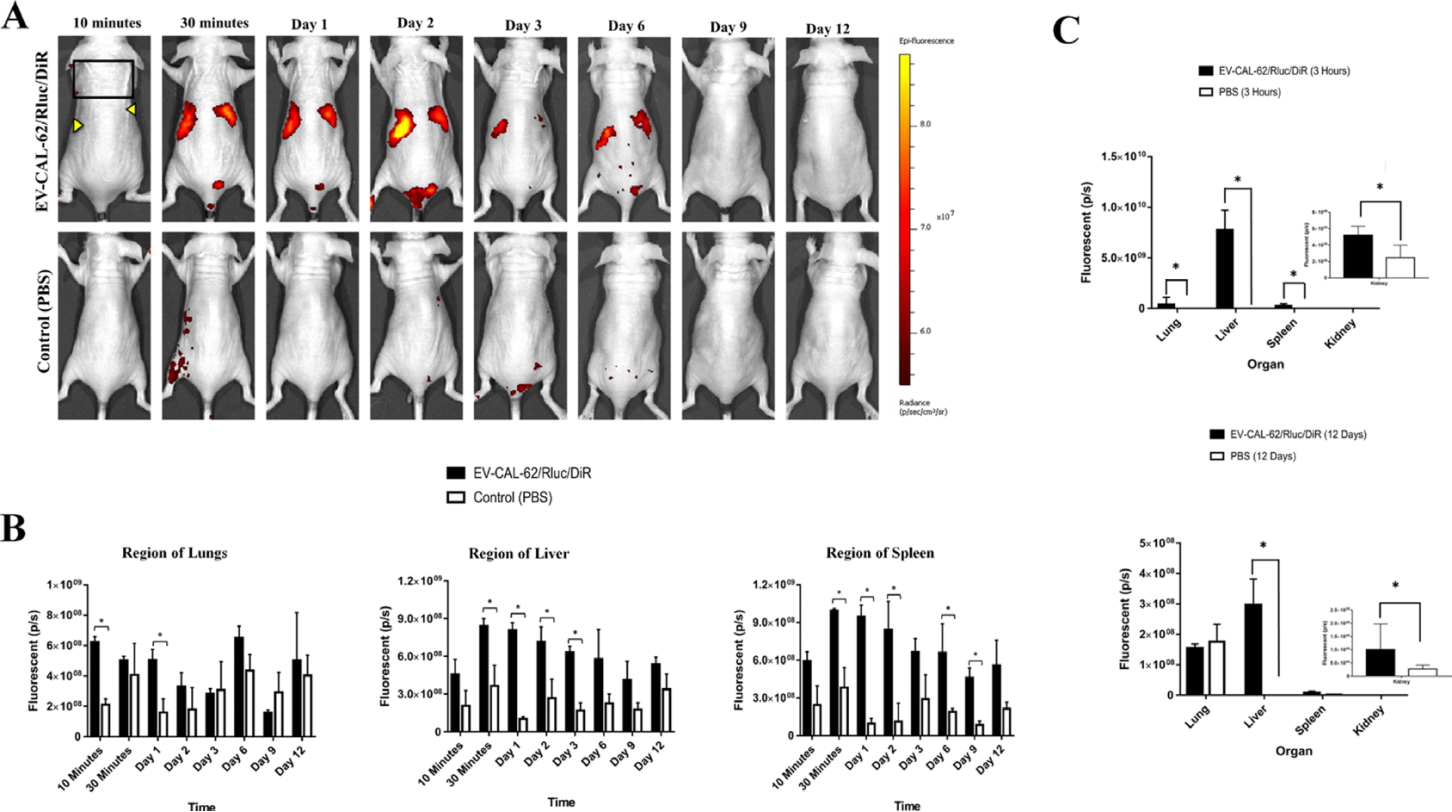
*In vivo* noninvasive fluorescent visualization of EV-CAL-62/Rluc/DiR biodistribution in nude mice and organ distribution (**A**) Representative *in vivo* fluorescent imaging (FLI) of EV-CAL-62/Rluc/DiR in nude mice. EV-CAL-62/Rluc/DiR or PBS (control) was administered via the tail vein. (**B**) Quantitation of EV-CAL-62/Rluc/DiR signal from regions corresponding to the lung, liver, and spleen after EV administration; the values are expressed as mean ± SD. ^*^P < 0.05 (Student's *t*-test). (**C**) Quantification of dissected organs of mice injected with EV-CAL-62/Rluc/DiR (*n* = 3) or PBS (*n* = 3) mice were euthanized at 3 hours and 12 days after injection. Florescence quantification in lungs, liver, spleen, and kidneys at 3 hours and 12 days (EV-CAL-62/Rluc/DiR or PBS). The values are expressed as mean ± SD, ^*^P < 0.05, (Student's *t*-test).

To assess the biodistribution of the EVs in mice by fluorescent imaging, the animals were imaged using an IVIS Lumina II. EV-CAL-62/Rluc/DiR was visualized in mice 30 minutes after injection. Fluorescent signals were predominantly detected in the regions of the liver and spleen, and little or no signal was detected in control mice (Figure 8A). The fluorescent signal intensity in the regions of the liver, lung, and spleen was quantified. At 10 minutes, no signal was detected with the IVIS, and at 30 minutes, a significantly strong fluorescent signal was detected in the regions of the liver and spleen (*P* < 0.05; Figure 8A, 8B). The strongest signal (*P* < 0.05) was detected and quantified in the region of spleen than in other organs. Quantification results revealed significantly stronger signals in the region of the lungs at 10 minutes and on day 1 (*P* < 0.05; Figure 8B). EV-CAL-62/Rluc/Dir were not visible in the animals by Day 9 post-injection (Figure 8B). Quantified results showed that little or no signal was detected on Day 12 (Figure 8B). Furthermore, on Day 3, the BLI signal was suddenly found to decline in the region of the liver and spleen (Figure 8B). The fluorescent signal in the region of the liver was significantly higher compared to control until Day 3 (*P* < 0.05), and fluorescent imaging data in the region of the spleen showed a statistically significantly higher signal until Day 9 (*P* < 0.05); relative to Day 3, there was a substantial increase (*P* = 0.083; Figure 8B). These experiments were designed to confirm the findings from *in vivo* EV-CAL-62/Rluc/DiR imaging and further analyze the organs from which the fluorescent and BLI signal originally originated. To accurately identify the organ from which the BLI signal originated, organs were collected at two time points (3 hours and 12 days) after EV administration, to assess DiR and Rluc signals. Fluorescent signals were significant after 3 hours in mice injected with EV-CAL-62/Rluc/DiR, in the liver (*P* < 0.05), followed by lungs (*P* < 0.05), spleen (*P* < 0.05), and kidneys (*P* < 0.05; Figure 8C and Supplementary Figure 4A). Fluorescent signals of EVs were not statistically significant at 12 days after EV-CAL-62/Rluc/DiR injection compared to PBS control in lungs (*P* = 0.36) and spleen (*P* = 0.23; Figure 7C and Supplementary Figure 4A); whereas a statistically significant signal was detected in the liver and kidneys (*P* < 0.05; Figures 8C and Supplementary Figure 4A). To confirm the DiR findings from imaging is correlating with Rluc imaging, further analyzed the exercised organs as mentioned above. The BLI signals detected at 3 hours, showed significantly stronger signals in liver compare to control (*P* < 0.05; Supplementary Figure 4B–4C). The higher signals were detected in liver followed by lungs, spleen and kidney (*P* < 0.05; Supplementary Figure 4B–4C). At 24 hours, no statistically significant signals were detected in all organs (Supplementary Figure 4B–4C). This result confirms that DiR labelling could change the distribution pattern of EVs *in vivo*. The immunofluorescent imaging of EV-CAL-62/Rluc/DiR injected mice's organ (3 hours after injection) revealed that most of the EVs co-localized with F4/80, which is biomarker for macrophages (Supplementary Figure 5A, 5B). Furthermore, some of the EVs are present other than macrophages, which revealed that the EVs either internalized to other cells present in the organ or localized in extracellular space and control (PBS) mice showed no DiR signals (Supplementary Figure 5A, 5B).

### EV-CAL-62/Rluc released into blood stream and spontaneously distributed to lung and liver, spleen and kidney in mice from subcutaneous CAL-62/Rluc tumor

**Figure 9:**
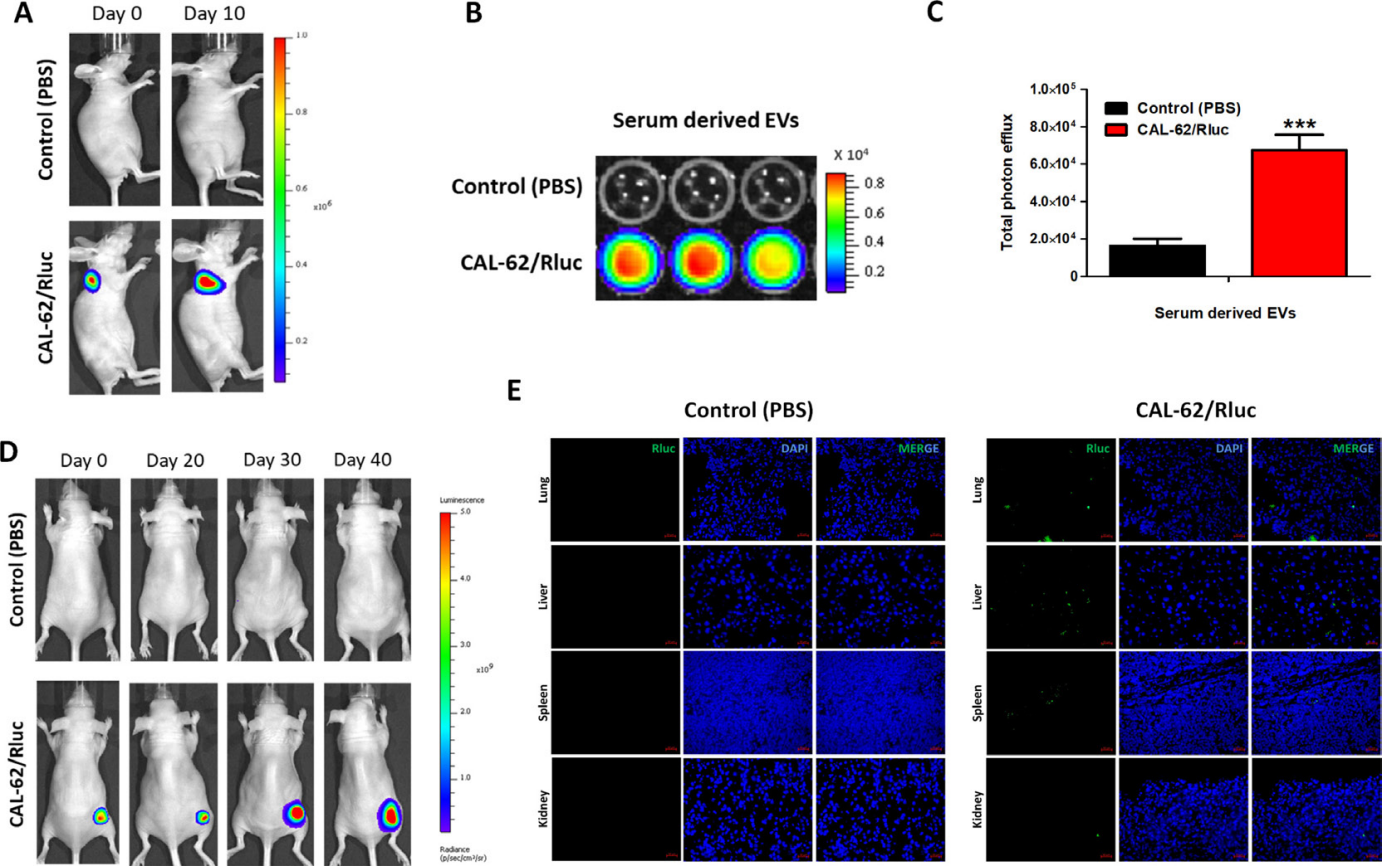
A spontaneous distribution of EV-CAL-62/Rluc in subcutaneous CAL-62/Rluc bearing mice (**A**) Representative image of Rluc activity of CAL-62/Rluc or PBS injected nude mice of day 0 and day 20. (**B**) Activity of Rluc in EVs isolated from the blood serum mice mentioned in (A). (**C**) Quantification of Serum derived EVs from CAL-62/Rluc (*n* = 3) or PBS (*n* = 3) mice. The values are expressed as mean ± SD, ^*^P < 0.05, (Student's *t*-test). (**D**) Representative image of Rluc activity of CAL-62/Rluc or PBS injected nude mice of day 0, 20, 30 and 40. (**E**) Representative confocal images of Rluc in harvested major organs (lung, liver, spleen and kidney) from mice mentioned in (D) at day 40. Scale bars: 50 μm.

*In vivo* bioluminescent imaging of systematically injected EV-CAL-62/Rluc showed that EVs localized to lung higher than the liver, spleen and kidney (Figure 5A–5C). To confirm whether the EV-CAL-Rluc settled down in lung due to route of injection or does this really have organ-tropism to lung. CAL-62/Rluc tumor bearing mice (Figure 9A) serum derived EVs (which includes the EV-CAL-62/Rluc); BLI imaging showed that significantly higher signal (*P* < 0.05; Figure 9B, 9C) was detected in CAL-62/Rluc tumor bearing mice compare to control (PBS injected) mice. Furthermore, mice injected with CAL-62/Rluc allowed growing up to 40 days (Figure 9D) and organs were removed and immunofluorescent staining with Rluc specific antibody, which revealed that EV-CAL-62/Rluc distributed to lung and liver much higher compare to spleen and kidney (Figure 9E). H&E staining revealed that no metastatic tumor cells were present in organs, which was confirmed by two pathologists (Supplementary Figure 6).

## DISCUSSION

Cell-to-cell communication is a dynamic process that enables cellular activities. Recent studies have shown that EVs released by different cell types may act as a mediator of cell-to-cell communication. Cancer development and progression depend on intercellular communication both locally and at a distance. Numerous studies have indicated that tumor-derived EVs are mediators of intercellular communication by evoking multiple biological responses like a protumorigenic response, proliferation, cell movement, and stem-ness leading to metastasis [20, 48, 49]. Apart from tumor EVs, dendritic cell (DC) exosomes are currently evaluated in clinical trials (e.g., trial # NCT01159288) as cancer treatment, and exosomes are evaluated for delivery of curcumin to colon cancer tissue (clinical trial # NCT01294072) [50]. Mesenchymal stromal cell-generated exosomes enhance the tissue regeneration after treatment [51, 52]. Thus, the direct visualization of tumor-derived EVs *in vivo* is urgently needed to understand the role of EVs in pathophysiologic processes and to develop theranostic strategies based on EVs.

Nonetheless, visualizing EVs released by cells has been challenging, therefore requiring a highly sensitive imaging method. Nonetheless, there are several methods of labeling and tracking EVs, such as direct labeling EV with a lipophilic dye or indirect labeling [26, 27, 32] and labeling with BLI reporter [29, 31, 35]. To date, tracking of EVs *in vivo* has required covalent linkage of the dye to EVs, membrane labeling with lipophilic dyes, or loading the cell with lipophilic dyes then isolation of EVs, all of which have certain limitations including the need to label the EVs immediately prior to injection. The signal generated by the directly labeled EVs yields stronger background and aggregation occurs during the labeling procedure [26, 27]. Other studies with visualization of EV did not test whether the differences in EV characteristics (distribution, morphology, concentration, and contents) are due to labeling with a dye or BLI reporter [16, 27, 29, 31, 32, 35]. Current EV-labeling methods have some limitations. Optical imaging using photons, both bioluminescent and fluorescent signals, is known to have the limitation of penetration in *in vivo* studies, especially for large animals. Therefore, quantification of the signal might also be intrinsically inaccurate. Nevertheless, quantification of the optical signals in small animals, such as mice, is widely used due to the relatively short signal path. Position of the animal during image acquisition can influence signal intensity of the optical signal by changing the overlying tissue thickness; therefore, we used exactly the same position for every mouse during BLI. In the current study, we developed a bioluminescent EV-labeling strategy to achieve live *in vivo* imaging of EVs in an animal model and compared the distribution pattern to EVs directly labeled with a dye (DiR).

Human ATC cells (CAL-62) and human breast cancer cell (MDA-MB-231) were stably transduced with Rluc lentivirus particles. EVs were prepared from a pre-purified culture medium derived from Rluc-expressing CAL62 and MDA-MB-231 cells. Western blot analysis confirmed the presence of EV marker proteins like ALIX and CD63. The presence of these markers is consistent with observations of purified EV samples, all EV preparations were free from contaminating cell organelles as indicated by the absence of markers of Golgi apparatus or endoplasmic reticulum [53, 54]. TEM analysis of the isolated EVs revealed the presence of vesicles with a characteristic round shape and a clear-cut lipid bilayer, showing good evidence of intact EVs. These results confirmed that the vesicles are derived from cells, in agreement with other studies [44, 55, 56]. Particle size distribution ranged from 30 to 500 nm by NTA. The isolated EVs showing the multiple peaks in NTA confirms the subpopulation (exosome and microvesicles) of our EVs. This quantification and size measurement revealed that many EVs have similar sizes, in line with results from other studies [36, 56].

*In vitro* imaging and western blotting of EVs derived from CAL-62/Rluc and MDA-MB-231 cells yielded evidence of the presence of the Rluc reporter protein in EVs, indicating selective distribution of the reporter protein in EVs, in good agreement with the results previously reported [29, 31, 35]. We observed the absence of Rluc mRNA in EVs derived from CAL-62/Rluc cells; the presence of functional Rluc mRNA within EVs will cause a longer signal in recipient organs *in vivo,* and it was ruled out by the mRNA assessment. It is known that mRNAs were not randomly secreted in EVs because diverse sequences were either preferentially secreted or, conversely, retained inside the cells [57]. Better knowledge of the mechanisms of biogenesis and secretion is still required. In this study, the release of Rluc from EVs was less than 2%. It was found that labeling of EVs with Rluc was stable in serum for 24 hours. Whereas our results showed an approximately 5% reduction in Rluc activity even in 24 hours. We confirmed that the Rluc protein present inside the EV compartment, which makes the Rluc protein, was not released outside EVs. Our Free Rluc activity assay results suggested that free form of Rluc showed a very shorter half-life compare to EV incorporated Rluc. These data suggest that labeling of EVs with Rluc has good stability, and the protein remained within EVs and was suitable for *in vivo* imaging and tracking of the EVs. Here, for the first time, we provide evidence that transduction of BLI reporter protein expression into CAL-62 and MDA-MB-231 cells does not significantly modify EV characteristics (distribution & concentration by NTA morphology by TEM, and protein contents by SDS-PAGE). These findings clearly suggest that the EVs had no major alterations other than Rluc protein reporter inside the EV compartment. A study showed increased internalization of exosomes by own cells [58]; this finding supports the use of cancer-cell EVs derived from patients for drug delivery. In addition, our cytotoxicity results suggest that EVs derived from the same cells or from patients’ own cells can be used for targeting the tumor. The targeting of own EVs must be studied thoroughly before clinical applications will become possible.

In the present study, we used nude mice for EVs biodistribution analysis because the most commonly used method for analysis of treatment of cancer involves immuno-deficient mice with a tumor xenograft. We intend to use tumor-derived EVs for delivery of a drug to the original tumor in a mouse tumor model. After i.v. administration of EV-CAL-62/Rluc or MDA-231/Rluc, the BLI first showed accumulation of signals in the region of the lungs. EV-CAL-62/Rluc predominantly localized in the lungs, whereas EV-MDA-231/Rluc localized both lung and liver. This finding is consistent with results of other studies [29, 35, 59, 60]. BLI signals of EV-CAL-62/Rluc that were observed in lungs were followed in strength by the liver and spleen. But the EV-MDA-231/Rluc signals were at lung and liver followed by Kidney and spleen. A few articles have also reported the distribution of exogenously administered EVs from HEK 293 cells *in vivo* [31, 35]; they were mostly detected in the liver followed by liver and lungs. Our results showed accumulation of the EVs in the region of lungs in case of EV-CAL-62/Rluc and; lung and liver in case of EV-MDA-231/Rluc. Our finding of accumulation of cancer-derived EVs in the lungs is consistent with two studies on EVs from a murine melanoma cell line [29, 35]. Furthermore, it was reported that EVs derived from breast and colon cancer cells aggregate in the liver and spleen followed by uptake in lungs and kidneys [32]. Our results showed EV-CAL-62/Rluc or EV-MDA-231/Rluc distributed differently in mice, which consistent with another studies with different tumor derived exosomes showed different distribution; in addition they also confirmed the integrins in exosome could be a reason for specific distribution to organs [60]. These results suggest that EVs derived from various cells seem to have different distribution, probably related to different contents and membrane composition, which are highly dependent on the cell origin.

In the current study, the BLI signal was detected 9 days and 3 days after administration of EV-CAL-62/Rluc and EV-MDA-231/Rluc respectively. Hence, the result suggests that the rate of tumor-derived EV clearance was not high as indicated in other reports [29, 31]. The slower uptake and clearance of EV-CAL62/Rluc was observed when compare to EV-MDA-231/Rluc. At the same time, previous report showed a slower clearance in mice with impaired innate immunity (nude mice) has been reported, which is consistent with our results [27]. Our results suggest that EVs are retained in the organ for a long period; this finding has not been reported before, to the best of our knowledge. Redistribution of EVs was detected between organs on Days 3 and 9 in EV-CAL-62/Rluc and day 3 in EV-MDA-231/Rluc (sudden increase of signal in kidney at day 3). The redistribution of EV was reported earlier [32]. Free Rluc protein administration to mice showed drastically different pattern of distribution compare to EV-CAL-62/Rluc and EV-MDA-231/Rluc. Furthermore, the Free Rluc protein retention time was shorter than EV-CAL-62/Rluc and EV-MDA-231/Rluc. This is very clear indication of the successful labeling of EV-CAL-62 and EV-MDA-231 with Rluc, other studies with Gluc failed to show the free Gluc protein biodistribution [29, 31]. Moreover the Rluc was not released from EV *in vivo*. This also indicates a good *in vivo* stability of Rluc inside the EV.

Furthermore, we have tested weather EVs distribution to internal organs due to route of injection or are specific to specific organs. Our results suggested that EV-CAL-62/Rluc released from the CAL-62/Rluc tumor bearing mice into blood stream. Recently, numerous studies used EVs as a biomarker for tumor diagnosis [61–63], which further confirms that EVs not completely eliminated immune cells in the tissue micro environment. Further immune-staining results suggested EV-CAL-62/Rluc were spontaneously distributed to internal organs, especially to lung and liver followed by spleen and kidney.

Further we verified weather the dye based direct labelling have any influence on *in vivo* distribution. After i.v. administration of EV-CAL-62/Rluc/DiR, the fluorescent signal was first observed in the region of the liver and spleen after 30 minutes, and no signal was detected at 10 minutes; In contrast, EV-CAL-62/Rluc/DiR results showed accumulation of the EVs in the region of the liver and spleen rather than in lungs when compared to our EV-CAL-62/Rluc-injected mice. BLI signals in EV-CAL-62/Rluc/DiR showed similar pattern to FLI signals which further confirms that DiR dye could influence the distribution of EVs *in vivo*. Twelve days after EV-CAL-62/Rluc/DiR injection, FLI signals showed signals in liver and kidney but BLI signals showed no signals at same time point. Which clearly explains that dye (DiR) can stay longer in organ [31] For instance, in two studies [29, 64], EVs derived from the same type of cells (melanoma) and the same administration routes were used, except labeling methods, but showed different organ distribution. In another study, DiI (NIR dye) and ^99m^Tc-HMPAO labeling showed different distribution patterns [65]. Further we showed subcellular visualization EV-CAL-62/Rluc/DiR injected organs by immunofluorescent, most of EVs were co-localized to macrophages and others cells in the specific organs, this admits the previous reports [60]. Cells appear to take up EVs by a variety of endocytic pathways, including, phagocytosis, and lipid raft-mediated internalization [66]. EVs are cleared mostly by diffusing inside the cells or released by renal route [31]. Our results also show comparatively higher signals in kidney compare to initially after EVs injection. We showed that the dye-based methods may influence the *in vivo* distribution of EVs compared to non-dye-based EVs. Furthermore, accurate tracking of EVs is limited in dye-based methods due to non-specificity of labeling and recirculation of the dye released from the EVs after their degradation [32]. Dye based labelling required an additional ultracentrifugation, which could damages the EVs structures and aggregation of the EVs [26, 67]. Moreover, extensive washing steps, needed to reduce the presence of dye residues which might result in nonspecific signals, can cause significant loss of the EVs [67]. EV organotropism is due to integrins present in the membrane of the EVs [60], which could be blocked by dye labelling at surface membrane of EVs leads to different distribution *in vivo*. The bioluminescence reporters can decipher the *in vivo* behavior of EVs with high sensitivity.

The visualization *in vivo* using the newly developed bioluminescent EVs with the reporter gene system is expected to serve as an invaluable tool for long-term, noninvasive EV-tracking experiments involving monitoring of EVs under physiological or pathological conditions. These goals have so far been unachievable by dye-based methods. In agreement with our *in vivo* findings, a significantly strong signal was detected in lungs followed by the liver, spleen, and kidneys, as reported earlier [27, 68]. A strong BLI signal was detected in the lung than in the liver. The kidney showed a bioluminescence signal, which could not be detected *in vivo*. Coelenterazine distributed well to most organs, including kidney. This is probably because the levels of EVs in the kidneys are lower and therefore below the detection threshold of the *in vivo* method or the organ location (depth of tissue) influences the intensity of BLI. This *ex vivo* experiment confirmed a different pattern of EV distribution as compared to BLI-EV (EV-CAL-62/Rluc). Our BLI of EVs (*in vivo* and *ex vivo*) overcomes the problems mentioned above (non-specificity of labeling or recirculation of retained fluorescence dyes after EV degradation). Multimodal tracking in the specific organs could also help to clearly detect the *in vivo* distribution of EVs.

A study on human subjects revealed that anaplastic thyroid cancer most commonly metastasizes to the lungs [69]. Tumor-derived EVs can induce cellular adhesion and spreading, due to rapid uptake and deposition of exosomes on the cell surface [70]. Cancer-derived EVs can contribute to the generation of pre-metastatic niches [71] and have proangiogenic properties [72], which may lead to stronger metastasis of the cancer in the lungs. The deposition of significantly more EVs in lungs may facilitate tumor growth at different metastatic sites, because numerous studies have shown that tumor-derived EVs can promote tumor progression [12, 24, 73]. Tumour derived exosome integrins determine the distribution of the specific organs, further these exosome prepare the organs to influx the circulating tumor cells leading to metastases in distal organs [60, 74]. EVs are in the ideal size-range for lymphatic transport. EVs are known distribute through the lymph node by lymphatic system [75, 76]. The role of lymphatic drainage and transport of EVs is as yet unknown, although the lymphatics play critical roles in immunity and tumor metastasis in lymph nodes. Further extensive studies in the future help us to understand the impacts of EVs on lymph node microenvironment and nodal metastases. Imaging and tracking of EVs could reveal that they are targeting specific tissues. Unfortunately, the existing studies are inconsistent depending on the methods/cell type/route of administration. Our results showing the distribution of tumor-derived EVs using the Rluc reporter system should prove to be useful for elucidation of the EV biology and for development of EV-based theranostic strategies.

## CONCLUSIONS

Here, for the first time, we report a study on *in vivo* BLI of EVs using an Rluc reporter system. The use of imaging reporter systems is gaining popularity for labeling of EVs for *in vivo* imaging, due to their accurate EV-associated signals. Using the approach to bioluminescent labeling of EVs described in this study, we have achieved visualization of EVs in an animal model and compared the data with the results of a dye-based labeling method. Our approach takes advantage of luciferase's dynamic range that covers an extensive spectrum of signal intensities, both *in vitro* and *in vivo*. We also demonstrated the biodistribution of the tumor-derived EVs, after intravenous injection into mice and spontaneous distribution of EVs *in vivo*. Tumor-derived EVs are rapidly distributed and retained in the lungs and liver, after which, they are cleared. There are few studies on dye-based labeling and bioluminescence labeling in EVs for *in vivo* imaging. Thus far, we demonstrated the quantitative capabilities of both luciferase-based bioluminescent and dye-based fluorescent *in vivo* imaging. Luciferase as a reporter has been shown to have various advantages that can be taken into consideration when choosing dye-based labeling or bioluminescence as the optical imaging modality, particularly for an EV distribution experiment. Therefore, our results should contribute to an improved understanding of the distribution of EVs in the mouse model. Real-time visualization of the fate of EVs as a function of time *in vivo*, which has been achieved in our study, is vital for evaluating the efficiency of EV-based delivery systems. In brief, we evaluated the visualization, distribution, tracking, and clearance of cancer-derived EVs *in vivo*. Moreover, our promising data support the feasibility of this approach for labeling of EVs with novel bioluminescent reporters. This study may advance the understanding of biodistribution of tumor-derived EVs and the development of EV-based delivery systems in emerging therapies.

## MATERIALS AND METHODS

### Cell culture

All cells were cultured in Dulbecco's Modified Essential Medium (DMEM; Gibco), supplemented with 10% of fetal bovine serum (FBS; Hyclone) and 1% penicillin-streptomycin (Gibco), at 37°C in an atmosphere containing 5% of CO_2_. CAL-62 and MDA-MB-231 cells were transduced with a lentivirus expressing Rluc and a puromycin resistance gene under the control of the cytomegalovirus (CMV) promoter (Genecopoeia) (CAL-62/Rluc & MDA-MB-231/Rluc cells). The optimal lowest concentration (kill curve) of puromycin to kill 100% of untransfected cells was determined by incubation of CAL-62 and MDA-MB-231 cells, plated in 6-well plates, with various concentrations of puromycin ranging from 1 to 10 ng/mL. We used 6 ng/mL puromycin for selection of stable clones. The transfected cells were grown in this medium for 2 weeks before selection of stable clones with puromycin (Sigma-Aldrich).

### An Rluc activity assay for CAL-62/Rluc and MDA-MB-231/Rluc cells

CAL-62 cells, CAL-62/Rluc cells, MDA-MB-231 and MDA-MB-231/Rluc (1.25 × 10^4^, 2.5 × 10^4^, 5 × 10^4^, and 10^5^ cells/well) were plated in white and clear-bottom 96-well plates with a serum-free DMEM medium. Twenty-four hours later, the appropriate substrate coelenterazine (Caliper, PerkinElmer) was added to each well. Rluc activity was determined by BLI, using the IVIS Lumina III instrument (*In Vivo* Imaging System, IVIS Lumina III, PerkinElmer).

### RT-PCR analysis

CAL-62 cells, CAL-62/Rluc cells, MDA-MB-231 and MDA-MB-231/Rluc cells and their respective EVs were lysed using a TRIzol solution (Invitrogen), and total RNA was extracted according to the manufacturer's instructions. Reverse transcription was performed as described elsewhere [77] using the RevertAid First Strand cDNA Synthesis kit (Fermentas). After denaturation of the samples for 2 minutes at 94°C, 40 cycles of 20 s at 94°C, 10 s at 57°C, and 30 s at 72°C were followed with an additional step of 5 minutes at 72°C. i-Taq DNA polymerase (iNtRON Biotechnology) and the GeneAmp PCR system were used. The primers were as follows: Rluc gene, forward, (5′-TATGAT TCCGAGAAGCACGC-3′, reverse, 5′-TGATCCAGGA GGCGATATGA-3′); and GAPDH (forward: 5′-AGTGATG GCATGGACTGTGG-3′; reverse: 5′-GTCAAGGCTGAG AACGGGAA-3′). The samples were separated by electrophoresis in an ethidium bromide-stained agarose gel. Gels were imaged on a UV transilluminator using a UVP GelDoc-IT imaging system.

### EV production and isolation

CAL-62 cells, CAL-62/Rluc cells, MDA-MB-231 and MDA-MB-231/Rluc cells were cultured in DMEM as described above, but EV-depleted FBS was used for all further EV production procedures. FBS was passed through a 0.22 μm syringe filter and centrifuged for 18 hours (overnight) at 120,000 × *g* at 4°C [78]. EVs were isolated according to a slightly modified Current Protocol in Cell Biology [44]. Briefly, after 3–4 days of cell culture, the supernatant was collected and sequential centrifugation was performed. The supernatant was first centrifuged at 300 × *g* for 10 minutes to remove live cells, and then at 1500 × *g* for 20 minutes to remove cell debris, and finally at 2500 × *g* for 20 minutes to remove apoptotic bodies. The supernatant was passed through a 0.45 μm syringe filter and centrifuged at 100,000 × *g* for 60 minutes. The EV pellets were then washed by resuspension in phosphate-buffered saline (PBS) to remove extracellular proteins and ultracentrifuged at 100,000 × *g* for 60 minutes. The final pellet was resuspended in 50–100 μl of PBS, stored at –20°C, and used within 7 days. All ultracentrifugation steps were performed (SW28 rotor; Ultra-Clear tube) using the Optima™ L-100 XP ultracentrifuge (Beckman Coulter). All centrifugation procedures were conducted at 4°C.

### Western blot analysis

Whole cells and EVs were treated with radio immune-precipitation assay (RIPA) buffer (Thermo Scientific) with a protease inhibitors cocktail (ATTO), and vortexed three times for 10 minutes, at 5-minute intervals. Subsequently, the sample was spun at 12,000 × *g* for 20 minutes, and the supernatant was collected. The protein yield was measured using the BCA Protein Assay Kit (Thermo Scientific). EVs membrane and cytosolic proteins were isolated according to the manufacture protocol (Thermo Fisher Scientific). Twenty micrograms of protein was loaded and separated by electrophoresis in denaturing SDS 10% polyacrylamide gels. The proteins were transferred from the gel to polyvinylidene difluoride (PVDF) membranes (Millipore), and the membranes were blocked with 5% Milk in Tris-buffered saline (TBS) for 1 hour and probed with the primary antibody overnight at 4°C, after three washes with TBS containing Tween 20 (TBST). Blots were incubated for 1 hour with a secondary antibody conjugated with horseradish peroxidase (HRP), in TBS with 2.5% Milk. The blots were washed three times with TBST. The primary antibodies used were as follows: Rluc (GeneTex; working dilution 1:2000), CD63 (Abcam; dilution 1:2000), ALIX (Abcam; dilution 1:2000), GM130 (Abcam; dilution 1:4000), Calnexin (Abcam; dilution 1:4000), and β-actin (Santa Cruz Biotechnology; dilution 1:5000). Secondary antibodies: HRP-conjugated anti-mouse and anti-rabbit IgG antibodies (Cell signaling; 1:8000) were used according to the manufacturer's instructions. The signals were detected using the ECL detection system (Bionote).

### Cellular and EV protein staining

CAL-62, EV-CAL-62, CAL-62/Rluc, and EV-CAL-62/Rluc or MDA-231, EV-MDA-231, MDA-231/Rluc, and EV-MDA-231/Rluc proteins (20 μg) were loaded and separated by electrophoresis in SDS 10% polyacrylamide gels. The gel was stained with Imperial™ protein stain (PIERCE).

### Transmission electron microscopy (TEM)

The vesicular pellets were obtained by ultracentrifugation as described above. EVs were imaged by TEM. Briefly, after isolation, EVs were fixed at 4°C overnight: the fixative contained 2.5% glutaraldehyde in 0.01 M phosphate buffer with pH 7.4 (passed through 0.22 μm filters) and was washed with PBS. EVs were post-fixed in 1% OsO_4_ (Taab Laboratories Equipment, Ltd.) for 30 minutes. EV pellets were washed with distilled water and dehydrated with graded ethanol. EV pellets were negative-stained with 1% uranyl-acetate in 50% ethanol for 30 minutes, and embedded in Taab 812 (Taab), followed by polymerization at 60°C overnight and ultrasectioning for TEM. The ultrathin sections were examined, and images were captured by means of a HT 7700 transmission electron microscope (Hitachi), operated at 100 kV.

### NanoSight analysis

Measurements of size and concentration of EVs were performed with a nanoparticle tracking analysis (NTA) using NanoSight LM10 (Malvern), equipped with a sample chamber with a 640 nm laser and a Viton fluoroelastomer O-ring. EVs resuspended in PBS were further diluted 500-fold with PBS. The samples were injected into the sample chamber with sterile syringes until the liquid reached the tip of the opposite outlet without any air bubbles. All measurements were performed at room temperature in triplicate. The SEM values obtained by the NTA software correspond to the arithmetic values calculated from the sizes of all the particles analyzed by the software.

### Rluc activity and stability assay for EV-CAL-62/Rluc, EV-MDA-231/Rluc

EV samples were plated in white and clear-bottom 96-well plates, in increasing concentrations of EV-CAL-62, EV-CAL-62/Rluc, EV-MDA-231 and EV-MDA-231/Rluc (15, 20, 25, 30, and 35 μg) at the same volume. Next, 20 μg of EV-CAL-62/Rluc was incubated in 20% FBS in a PBS solution at 37°C [35]. Stability of Rluc was evaluated by measuring Rluc activity at 0, 6, 12, 18, 24 hours. The appropriate substrate coelenterazine was added to each well. Rluc activity was determined by BLI as described above.

### Rluc stability assay recombinant Rluc protein (Free Rluc)

Stability of Free Rluc protein (Creative BioMart) was evaluated by measuring Rluc activity at 0, 1, 2, 3, 4 and 5 hours. The appropriate substrate coelenterazine was added to each well. Rluc activity was determined by BLI as described above.

### Rluc-binding capacity of EVs in serum

Fifty microliters of EV-CAL-62/Rluc and EV-MDA-231/Rluc was diluted 100-fold with 4950 μl of 20% FBS in PBS containing 1% EDTA-free Protease Inhibitor Cocktail (ATTO). The diluted samples were ultracentrifuged at 100,000 × *g* for 1 hour at 4°C to pellet the EVs. Rluc activity in the supernatant was determined at 0, 6, 12, 18, 24 hours by BLI as described above.

### Proteinase K treatment assay

EV-CAL-62/Rluc and EV-MDA-231/Rluc purified by ultracentrifugation and resuspended in 20 μl PBS. Equal amount of Samples were incubated in either PBS or 10 μg/ml Proteinase K (Sigma) for 1 hour at 37°C [79]. Immediately after 1 hour, Rluc activity was measured by IVIS as described above.

### DiR-labeled EVs (EV-CAL-62/Rluc/DiR)

EV-CAL-62/Rluc isolated by the UC method as described above and EV pellets were incubated with 1 mM DiR (fluorescent lipophilic tracer; Invitrogen, Life Technologies) at room temperature (RT) for 15 minutes. EVs were washed with PBS twice to remove unbound DiR by ultracentrifugation.

### Bioluminescence and fluorescence imaging of EVs *in vivo*

Female BALB/c nude mice (5.5 weeks old) were obtained from Hamamatsu (Shizuoka). The mice were maintained for adaptation to experimental conditions for 10 days before initiation of the experiment. The animals were maintained at room temperature (20–25°C), and relative humidity was set to ∼40–70%. The mice were subdivided into two groups (*n* = 3). Freshly isolated 25 μg (in terms of protein) of EV-CAL-62/Rluc or EV-MDA-231/Rluc or EV-CAL-62/Rluc/DiR in PBS was intravenously injected through the tail vein. The mice were anesthetized with 2.5% isoflurane (Merial, Lyon), and for EV-CAL-62/Rluc images were acquired after 10 and 30 minutes, and then after 1, 2, 3, 6, 9, and 12 days after the EV CAL-62/Rluc or PBS injection; for EV-MDA-231/Rluc images were acquired after 10 and 30 minutes, and then after 1, 2, 3 and 6 days after the EV-MDA-231/Rluc or PBS injection, For BLI resulting from 150 μl of coelenterazine administration. For *in vivo* fluorescent imaging, the mice anesthetized with 2.5% isoflurane and images captured with IVIS Lumina III were used. The BLI and fluorescent signals were quantified in the lungs, liver and spleen kidney region (where ever possible) using the IVIS software (Living Image Software, PerkinElmer). All the animals were imaged at the same binning and/or capture time between experimental and control animals.

### *Ex vivo* tissue distribution of EV-CAL-62/Rluc, EV-MDA-231/Rluc and EV-CAL-62/Rluc/DiR

EV-CAL-62/Rluc or EV-MDA-231/Rluc or PBS was intravenously injected into nude mice through the tail vein. At 3 hours and 12 days for EV-CAL-62/Rluc and or At 3 hours and 6 days for EV-MDA-231/Rluc, animals (*n* = 3) were euthanized, organs were excised, and tissues were homogenized and lysed with Mammalian protein extraction reagent (Thermo Fisher Scientific) and protease inhibitor (ATTO). The homogenate was centrifuged at 12,000 × *g* for 20 minutes at 4°C. The protein yield was measured as described above. Next, 50 μg of protein from each organ was loaded into 96-well plates. Rluc activity was determined as described above. For *ex vivo* analysis of EV-CAL-62/Rluc/DiR, at 3 hours and 12 days, animals (*n* = 3) were euthanized, and organs were rapidly dissected and placed under IVIS Lumina III for analysis. Images were acquired and analyzed as described above.

### *In vivo* and *ex vivo* bioluminescence imaging of free Rluc protein

One μg of Free Rluc protein (Creative BioMart) diluted in PBS was intravenously injected through the tail vein of mice (*n* = 3) and PBS control (*n* = 3). The mice were anesthetized with 2.5% isoflurane, and images were acquired as mentioned above after 1, 10 and 30 minutes, and then after 1, 3 and 24 hours of post injection. Free Rluc or PBS was intravenously injected into nude mice through the tail vein. At 30 minutes and 24 hours animals (*n* = 3) were euthanized, organs were excised, and tissues were homogenized and lysed. Images were acquired and analyzed as described above.

### Establishing a CAL-62/Rluc subcutaneous tumor mice model and isolation of serum derived EVs

CAL-62/Rluc subcutaneous tumor was established in 6-week-old female nude mice by injection of 5 × 10^6^ cells into the upper right flank (*n* = 4) or lower right flank (*n* = 4) or PBS (*n* = 8). Tumor growth was assessed by was measuring Rluc activity by IVIS Lumina II as mentioned above. For Rluc activity, coelenterazine 20 μl (10 mg/5 ml ethanol) was mixed with sodium phosphate buffer and injected 200 μl per mouse by IV and immediately started the IVIS imaging. CAL-62/Rluc or PBS injected in upper right flank mice's (*n* = 4) venous blood were collected and serum were separated by centrifuge to isolate the EVs in serum. Serum EVs were isolated by ultracentrifuge and Rluc activity was measured as mentioned above. CAL-62/Rluc or PBS injected in lower right flank mice's (*n* = 4) were sacrificed at day 40 and organ such as lung, liver, spleen and kidney processed for staining assays.

### Immunofluorescence (IF) and histology

EV-CAL-62/Rluc/Dir or PBS injected mice after 3 hours mice organs were sectioned and IF assay performed as mentioned previously [4] with anti-F4/80 (rabbit) and Alexa Flora 488 goat anti-rabbit antibody. CAL-62/Rluc or PBS injected in lower right flank mice's (*n* = 4) sectioned organs were stained with anti-Rluc (rabbit) and Alexa Flora 488 goat anti-rabbit antibody. IF stained sections were imaged under confocal microscope (Zeiss, Germany). H&E staining was performed as described previously [80] on same sections.

### Statistical analysis

All data are expressed as mean ± standard deviation (SD), two groups of data were statistically analyzed by *t*-test using GraphPad Prism5 software, version 5.01 (GraphPad Software, Inc. USA). Differences with a *P* value less than 0.05 were considered statistically significant.

## SUPPLEMENTARY MATERIALS FIGURES


