# Correction: A new bioluminescent reporter system to study the biodistribution of systematically injected tumor-derived bioluminescent extracellular vesicles in mice

**DOI:** 10.18632/oncotarget.27473

**Published:** 2020-03-17

**Authors:** Prakash Gangadaran, Xiu Juan Li, Ho Won Lee, Ji Min Oh, Senthilkumar Kalimuthu, Ramya Lakshmi Rajendran, Seung Hyun Son, Se Hwan Baek, Thoudam Debraj Singh, Liya Zhu, Shin Young Jeong, Sang-Woo Lee, Jaetae Lee, Byeong-Cheol Ahn

**Affiliations:** ^1^ Department of Nuclear Medicine, Kyungpook National University School of Medicine and Hospital, Daegu 700-721, Republic of Korea; ^2^ Department of Medical Oncology, All India Institute of Medical Sciences (AIIMS), Ansari Nagar, New Delhi 110029, India

**This article has been corrected:** During the assembly of images for Figure 5, the image for Day 1 in the control (PBS) group, seen in panel A, was erroneously copied to Day 2 as well. The corrected Figure 5, obtained using the original data, is shown below. The authors declare that these corrections do not change the results or conclusions of this paper.

Original article: Oncotarget. 2017; 8:109894–109914. 109894-109914. https://doi.org/10.18632/oncotarget.22493

**Figure 5:**
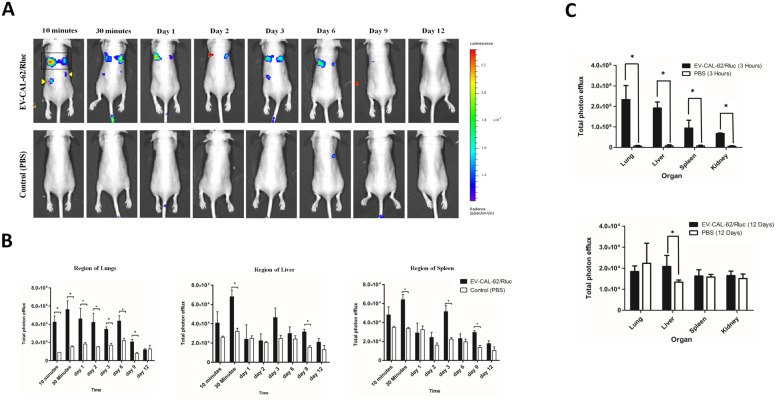
*In vivo* noninvasive bioluminescent visualization of EV-CAL-62/Rluc biodistribution in nude mice and organ distribution. (**A**) Representative *in vivo* bioluminescent imaging (BLI) of EV-CAL-62/Rluc in nude mice. EV-CAL-62/Rluc or PBS (control) was administered via the tail vein. Coelenterazine was injected via the same route at 10 and 30 min and 1, 2, 3, 6, 9, and 12 days after initial administration to visualize EV-CAL-62/Rluc. Regions of the lung (four-sided box), liver (right arrowhead), and spleen (left arrowhead) are indicated in the animal (at 10 minutes). (**B**) Quantitation of EV-CAL-62/Rluc signal from regions corresponding to the lung, liver, and spleen after EV administration; the values are expressed as mean ± SD. ^*^ *P* < 0.05 (Student’s *t*-test). (**C**) Quantification of dissected organs of mice injected with EV-CAL-62/Rluc (*n* = 3) or PBS (*n* = 3). The mice were euthanized at 3 hours and 12 days after injection. Organs were harvested and lysed, and Rluc activity was measured. Bioluminescence quantification of lungs, liver, spleen, and kidneys at 3 hours and 12 days (EV-CAL-62/Rluc or PBS); the values are expressed as mean ± SD, ^*^ *P* < 0.05, (Student’s *t*-test).

