# Supplementary material for: A new bioluminescent reporter system to study the biodistribution of systematically injected tumor-derived bioluminescent extracellular vesicles in mice
Source: Oncotarget. 2017 Nov 18;8(66):109894–914. doi: 10.18632/oncotarget.22493 (PMC5746352; doi:10.18632/oncotarget.22493)
Supplement: Supplementary file 1 [file oncotarget-08-109894-s001.pdf]

A new bioluminescent reporter system to study the biodistribution of systematically injected tumor-derived bioluminescent extracellular vesicles in mice

SUPPLEMENTARY MATERIALS

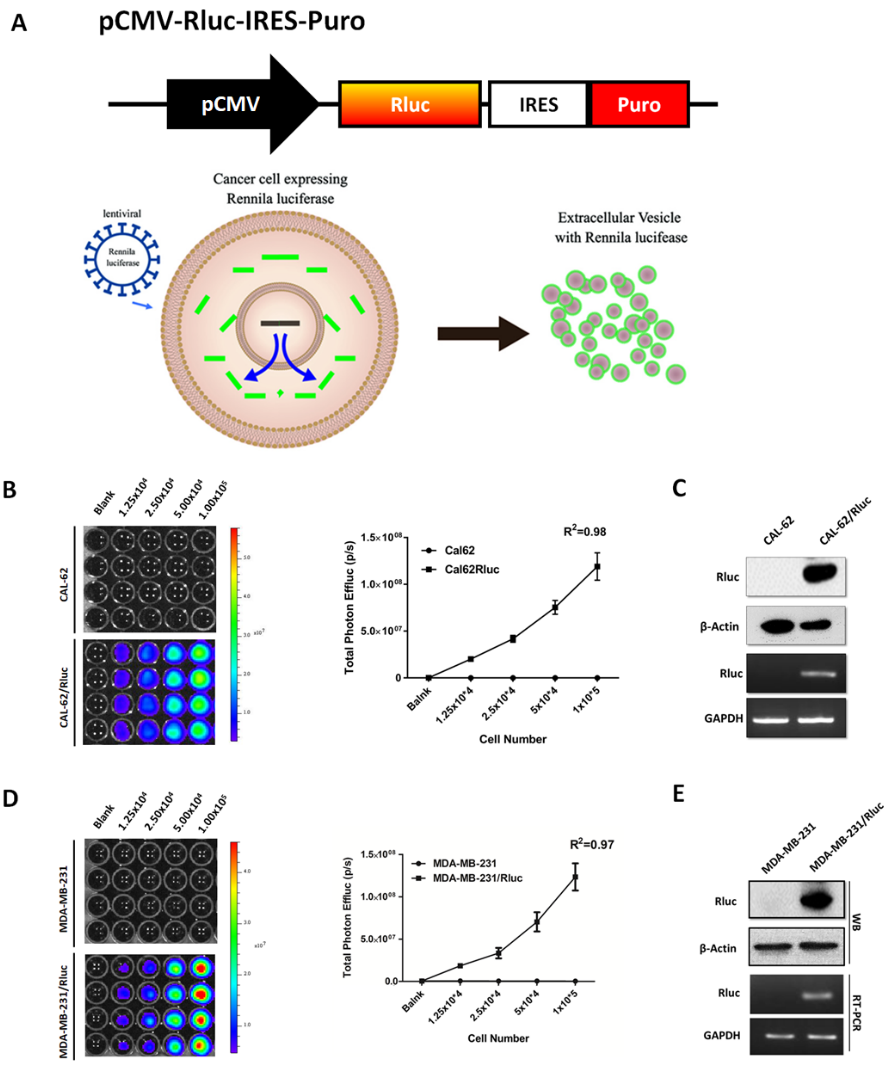

Supplementary Figure 1: Generation of stable reporter gene expression in the cancer cell line. (**A**) A diagrammatic representation of plasmid constructs, transfection and EV expressing Rluc. (**B**) Representative bioluminescent imaging of the *in vitro* luciferase assay in CAL-62 and CAL-62/Rluc cells. An *in vitro* luciferase assay in CAL-62 and CAL-62/Rluc cells. Data are expressed as mean ± standard deviation (SD). (**C**) Western blot analysis of the Rluc (37 kDa) protein in CAL-62/Rluc and CAL-62 cells; β-actin was used as an internal control. RT-PCR analysis to determine the expression of the *Rluc* gene in CAL-62/Rluc cells and CAL-62 cells; GAPDH served as an internal control. (**D**) Representative bioluminescent imaging of the *in vitro* luciferase assay in MDA-MB-231 and MDA-MB-231/Rluc cells. An *in vitro* luciferase assay in MDA-MB-231 and MDA-MB-231/Rluc cells. Data are expressed as mean ± standard deviation (SD). (**E**) Western blot analysis of the Rluc (37 kDa) protein in MDA-MB-231/Rluc and MDA-MB-231 cells; β-actin was used as an internal control. RT-PCR analysis to determine the expression of the *Rluc* gene in MDA-MB-231/Rluc cells and MDA-MB-231 cells; GAPDH served as an internal control.

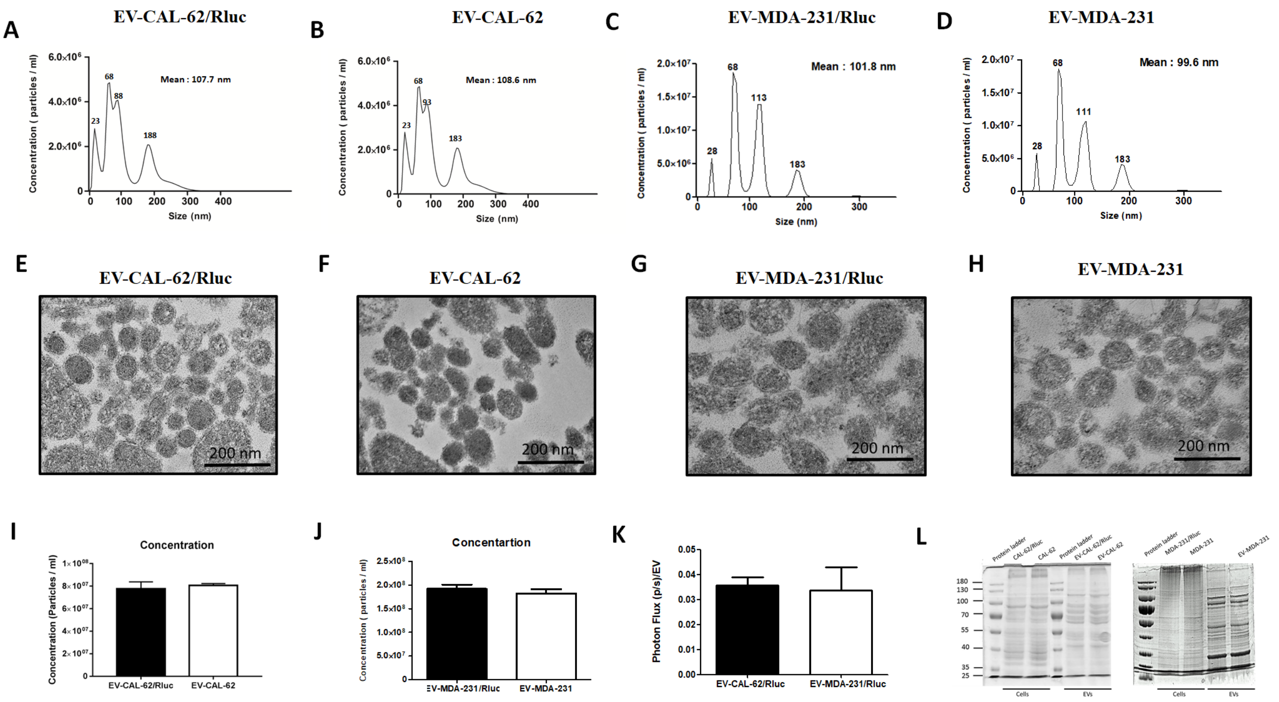

Supplementary Figure 2 : EV size and structure are not affected by Rluc manipulations in cells. (**A**–**D**) Size of EVs derived from by CAL-62/Rluc, CAL-62 cells, MDA-MB-231/Rluc and MDA-MB-231 as measured by NanoSight. (**E**–**H**) Electron-microscopic examination of EVs from CAL-62/Rluc, CAL-62 cells, MDA-MB-231/Rluc and MDA-MB-231. (**I**, **J**) Concentration of EVs from CAL-62/Rluc, CAL-62 cells, MDA-MB-231/Rluc and MDA-MB-231 were measured. Sizes of EVs did not differ significantly (CAL-62: $P = 0.22$ and MDA-MB-231: $P = 0.42$). The concentration is expressed as the number of particles per milliliter. Data are expressed as mean ± SD. (**K**) The Rluc expression (Photon Flux) in a single EVs of CAL-62/Rluc and MDA-MB-231/Rluc are expressed as mean ± SD. (**L**) Detection of EV proteins derived from the CAL-62/Rluc, CAL-62 MDA-231/Rluc and MDA-231 cells and their respective EVs by Coomassie brilliant blue (CBB) staining.

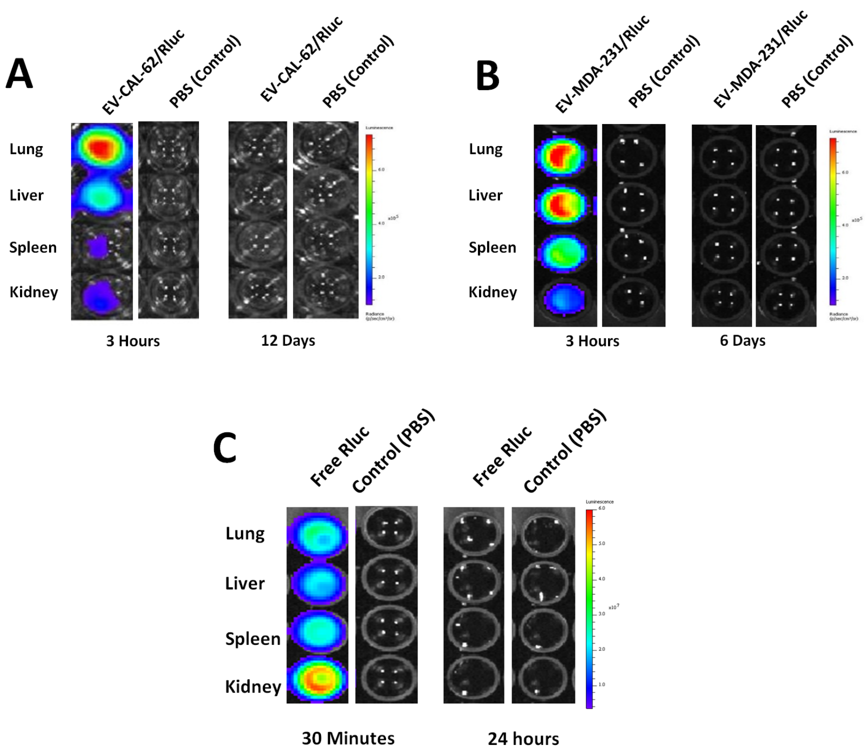

Supplementary Figure 3: Biodistribution of i.v. administered EV-CAL-62/Rluc, EV-MDA-231/Rluc and free Rluc in organs. (**A**) Representative *in vivo* bioluminescent imaging (BLI) of dissected organs of mice injected with EV-CAL-62/Rluc ($n = 3$) or PBS ($n = 3$); the mice were euthanized at 3 hours and 12 days after injection. (**B**) Representative *in vivo* bioluminescent imaging (BLI) of dissected organs of mice injected with EV-MDA-231/Rluc ($n = 3$) or PBS ($n = 3$); the mice were euthanized at 3 hours and 6 days after injection. (**C**) Representative *in vivo* bioluminescent imaging (BLI) of dissected organs of mice injected with Free Rluc ($n = 3$) or PBS ($n = 3$); the mice were euthanized at 30 minutes hours and 24 hours after injection.

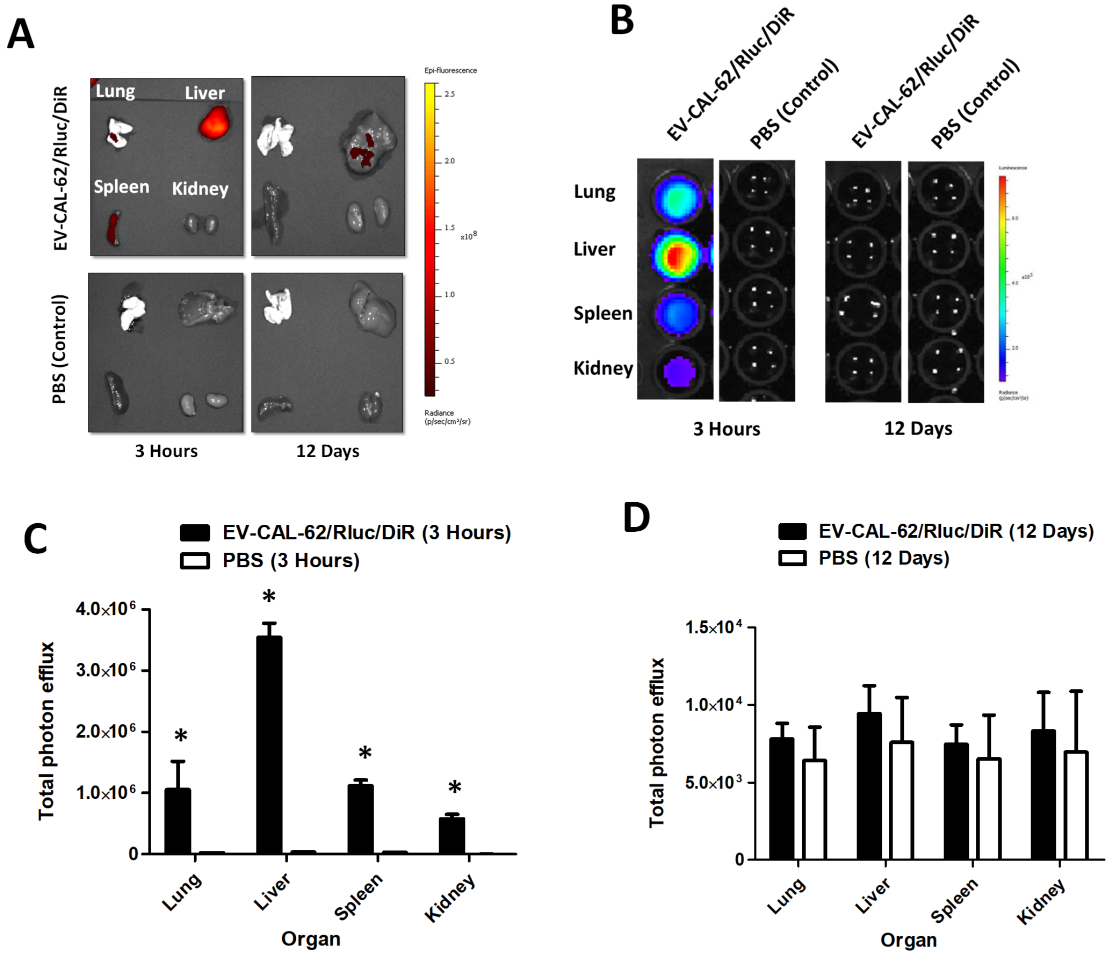

Supplementary Figure 4: Biodistribution of i.v. administered EV-CAL-62/Rluc/DiR in organs. (**A**, **B**) Representative in vivo fluorescent imaging (FLI) and bioluminescent imaging (BLI) of dissected organs of mice injected with EV-CAL-62/Rluc/DiR (n = 3) or PBS (n = 3) mice were euthanized at 3 hours and 12 days after injection. (**C**, **D**) Bioluminescence quantification of lungs, liver, spleen, and kidneys at 3 hours and 12 days (EV-CAL-62/Rluc or PBS); the values are expressed as mean ± SD, $^{*}P < 0.05$, (Student's t-test).

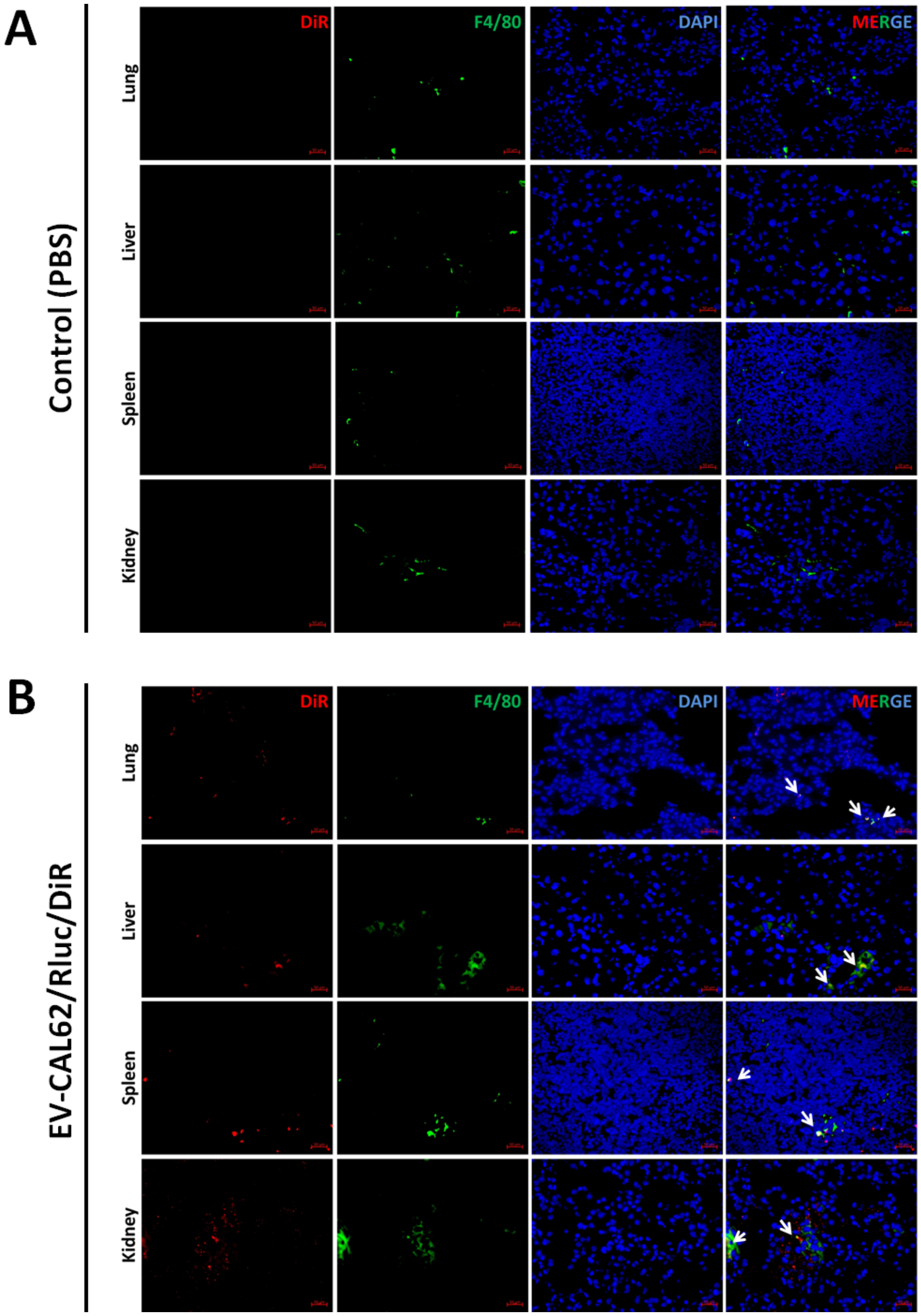

Supplementary Figure 5: Subcellular visualization of i.v. administered EV-CAL-62/Rluc/DiR in organ. (**A**, **B**) Mice injected with EV-CAL-62/Rluc/DiR (n = 3) or PBS (n = 3) mice were euthanized at 3 hours after injection. Cryo-sectioned and immunostained with anti-F4/80 (rabbit) and Alexa Fluor 488 goat anti-rabbit antibodies. EV-CAL-62/Rluc/DiR (arrow) was co-localized with macrophages cells in organs. Nuclei were visualized by 4,6-diamidino-2-phenylindole (DAPI). Bar, 50 µm.

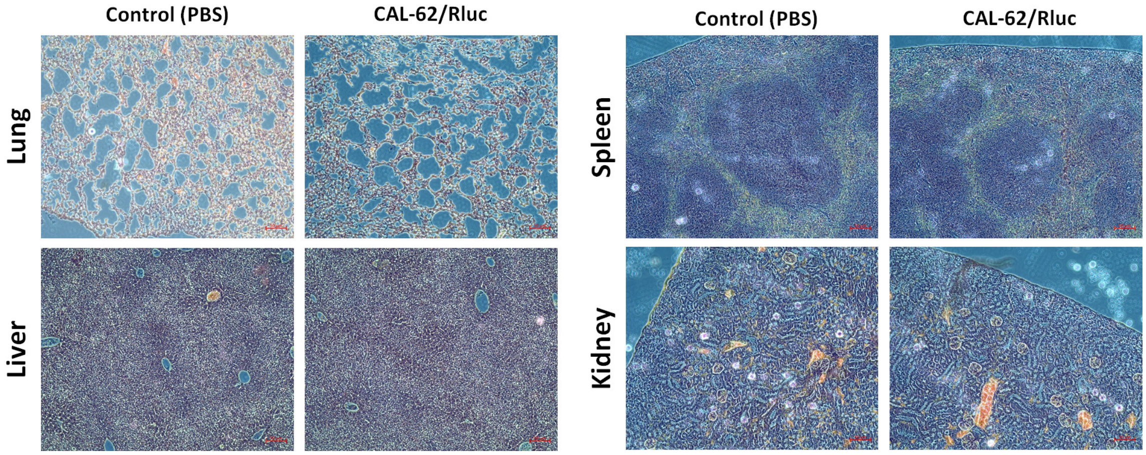

Supplementary Figure 6: Microscopic examination of organs isolated from CAL-62/Rluc tumor bearing and Control (PBS) mice. Lung, liver, splenic, and kidney tissues were stained with H&E. Scale bar: 50 µm.
